# The efficacy of nanoparticles on soil microbial biodiversity and the prevention of Fusarium wilt disease (*Fusarium oxysporum f.sp. lycopersici*)

**DOI:** 10.1186/s12866-025-04022-3

**Published:** 2025-05-19

**Authors:** Tahsin Shoala, Hoda A. S. El-Garhy, Nevein A. S. Messiha, Sozan E. El-Abeid

**Affiliations:** 1 Environmental Biotechnology Department, College of Biotechnology, Misr University for Science and Technology, Giza, 12563 Egypt; 2 Genetics and Genetic Engineering Dept, Faculty of Agriculture, Benha University, Qalyubia, Egypt; 3 Bacterial Disease Research Department, Plant Pathology Research Institute, Agricultural Research Center (ARC), Giza, Egypt; 4 Nanotechnology & Advanced Nano-Materials Laboratory (NANML), Mycology and Disease Survey Research Department, Plant Pathology Research Institute, Agricultural Research Center (ARC), Giza, 12619 Egypt

**Keywords:** *Fusarium oxysporum f*.sp. *Lycopersici*, Salicylic acid nanoparticles (SA-NPs), Glycyrrhizic acid nanoparticles (GAS-NPs), Beneficial bacteria, PR genes

## Abstract

*Fusarium oxysporum f.sp. lycopersici (FOL)* wilt endangers Egyptian tomato productivity. Nanotechnology has emerged as an efficient tool for managing plant diseases. This study evaluated salicylic acid nanoparticles (SA-NPs) and glycyrrhizic acid ammonium salt nanoparticles (GAS-NPs) against *F. oxysporum in vitro.* SA-NPs reduced *F. oxysporum* growth by 37.8%, and GAS-NPs by 18.9% at 3 ml/L, while SA-NPs at high doses significantly reduced the bacterial count in the tomato rhizosphere. Under greenhouse conditions, high doses of SA-NPs suppressed disease by 73%, compared to 87-93% for other treatments, coinciding with a significant decrease in the overall bacterial count in the tomato rhizosphere. A high dose of SA-NPs reduced heterotrophic, copiotrophic, and fluorescent pseudomonads in the tomato rhizosphere but did not affect the total number of fungi. In vitro, a high dose of both nanoparticles did not significantly reduce bacterial growth in four tested strains: *Leclercia adecarboxylata*, *Pseudomonas putida*, *Enterobacter ludwigii*, and *Bacillus marcorestinctum*. This suggests that while SA-NP doesn’t directly affect bacterial growth, it may interact with tomato roots, indirectly affecting the rhizosphere bacterial population. All treatments increased the expression of ethylene-responsive transcription factor 3 (RAP), xyloglucan endotransglucosylase 2 (XET-2), catalytic hydrolase-2 (ACS-2), phenylalanine ammonia-lyase 5 (PAL5), lipoxygenase D (LOXD), proteinase inhibitor II (PINII), and pathogenesis-related protein 1 (PR1). The highest gene expression levels were obtained from 1 ml/L GAS-NPs and SA-NPs field applications. Furthermore, SA-NPs at 1 ml/L were the most efficient in controlling tomato Fusarium wilt, followed by GAS-NPs. This study investigates the possibility of nanotechnology-based techniques for decreasing Fusarium wilt in tomatoes. However, because of the deleterious impact on the soil bacterial community, high dosages of NPs, particularly SA-NPs, should be applied with caution. Future research should focus on optimizing NPs doses to maintain a balance between efficient disease control and the maintenance of the beneficial complexity of soil microbial biodiversity.

**Supplementary Information:**

The online version contains supplementary material available at 10.1186/s12866-025-04022-3.

## Introduction

Tomato fruits (*Lycopersicon esculentum* Mill) are essential crops in Egypt and other countries. However, tomato plants in Egypt face a severe threat from Fusarium wilt, a soil-borne pathogen that can cause serious vascular diseases, rot, and damping-off diseases in various crops. The disease, first documented in Europe in 1933 [1], this disease demonstrates high adaptability and diversity. Fusarium strains have high host specificity and saprophytic features, allowing them to persist in the soil and rhizosphere for extended periods [2]. Defense mechanisms in plants involve the rapid creation of antioxidants and changes in cell wall composition, which stimulate pathogen defense gene expression [3, 4]. Fusarium toxins are the most common natural pollutants found in cereals and grains, and their toxic qualities have been investigated for possible health hazards [5].

Conventional approaches, such as cultural, physical, and chemical for managing Fusarium wilt, these methods, have proven ineffectual [6], while chemical fungicides raised environmental concerns. Techniques like fumigation and the cultivation of resistant varieties have shown limited success, as resistant types still exhibit lower host vascular colonization compared to susceptible plants [7, 8].

Nanotechnology emerges as a promising solution to address these limitations. This rapidly advancing field has numerous applications in agriculture and medicine. In particular, Nanotechnology can save, improve, and protect crops and livestock when integrated with natural resources. Nanoparticles (NPs) have recently sparked widespread interest due to their biocompatibility, low toxicity, and environmental sustainability [9]. Nanotechnology has shown growing potential in managing plant diseases [10]. NPs can function as direct plant defenses or pesticide transporters [11, 12]. Because of their size-dependent features, high surface-to-volume ratio, and distinct optical properties, nanomaterials hold great promise for applications in fertilizing and plant protection. Studies have shown that nanotechnology can be used in plants [13]. Nano-sized mineral compositions improve the solubility, dispersion, and absorption of previously insoluble nutrients in soil. They also minimize soil fixation and increase the bioavailability of nanostructured particles. As a result, nano-nutrient elements (non-fertilizers) help to improve nutrient efficiency and plant uptake [14]. For example, chitosan-lactide copolymer nanoparticles have been found to enhance the solubility and efficiency of low-solubility fungicides like pyraclostrobin [15]. However, it is important to note that much of the study in this sector has taken place in laboratories [16].

Natural transformation products at the nanoscale can efficiently control phytopathogenic fungus without affecting the environment or human health [17]. The nanoactivity of natural compounds against phytopathogens has promised a variety of applications, including increasing the shelf life of fruits and vegetables at room temperature. Furthermore, it can minimize pesticide effects and toxicity while minimizing the possible detrimental effects of undiscovered nanoparticles [18, 19].

Salicylic acid (SA) is an important signaling molecule involved in a wide range of biotic and abiotic stress responses. It is crucial to the processes of plant development. A couple of its primary processes aid in the adaptation to environmental stress [20]. Furthermore, glycyrrhizic acid (GA), a bioactive molecule found in licorice roots and rhizomes [21], has long been used as a natural cure for fungal infections [22], and antioxidants [23].

The most efficient method of protecting seeds and leaflets may involve utilizing nanoparticles to resist invading diseases. In this setting, nanoparticles (NPs) can perform similarly to synthetic insecticides. However, it is critical to evaluate the larger consequences of nanoparticle use in soil, particularly their effects on non-target organisms. This issue is especially concerning when non-target species play important roles in soil ecosystems. A previous treatment primes plant defenses, increasing resistance to recurring pathogen infections. This is known as systemic acquired resistance (SAR) [24]. Molecular genetic studies on tomato plant resistance have significantly advanced our understanding of the plant genetic mechanisms against different pathogens [25, 26]. Even though little is known about the molecular features of the signaling mechanism [ 27]. Previous research has shown that biotic and abiotic stressors regulate the genes proteinase inhibitor II (PINII), phenylalanine ammonia-lyase 5 (PAL5), lipoxygenase D (LOXD), xyloglucan endotransglucosylase 2 (XET-2), catalytic hydrolase-2 (ACS-2), ethylene-responsive transcription factor 3 (RAP), and pathogenesis-related protein 1 (PR1). Plant SAR mechanisms include the ethylene, salicylic acid, and jasmonic acid pathways, to which these seven defense genes belong [28–30].

There have been few investigations on the effects of nanoparticles on soil health. AgNP exposure had a major impact on the soil microbial community, resulting in large microbial biomass decreases and changes in community composition. For example, populations of bacterial ammonia oxidizers and beta-Proteobacteria declined, whereas Acidobacteria, Actinobacteria, and Bacteroidetes grew [31]. Meanwhile, the use of SA-NPs and GAS-NPs in agriculture is advocated. AgNPs break microbial cell membranes and produce reactive oxygen species (ROS) [32], but SA-NPs and GAS-NPs improve plant defense systems and have anti-inflammatory properties.

The purpose of this study is to determine the efficacy of salicylic acid nanoparticles (SA-NPs) and glycyrrhizic acid ammonium salt nanoparticles (GAS-NPs) in the treatment of *Fusarium oxysporum*-induced fungal wilt and on tomato plants growth and yielding. The study also investigates the influence of these nanoparticles on soil-culturable bacteria and fungi, as well as how they affect the growth of some beneficial bacteria. Finally, by assessing the expression of RAP, XET-2, ACS-2, PINII, PAL5, LOXD, and PR1, the important genes of the ethylene, jasmonate, and salicylic acid pathways, we sought to see whether we could identify the induction of SAR-related defence genes.

## Materials and methods

### Materials

#### SA and GA synthesis and characterization

A 0.2 mg sample of Salicylic acid (SA) (Sigma-Aldrich Company, CAS Number: 69-72-7) according to (Amin et al., 2008) was dissolved in 1 ml of absolute ethanol and sonicated (XUBA3 Analogue Ultrasonic Bath, Grant Company) with an ultrasonic power and frequency of 50 kHz for one hour at room temperature (25 °C). A 0.1 mg sample of Glycyrrhizic acid ammonium salt (GAS) (Sigma-Aldrich, CAS Number: 53956-04-0) was dissolved in 1 ml of absolute ethanol and sonicated in an XUBA3 Analogue Ultrasonic Bath (Grant Company) for one hour at ambient temperature (25 °C) using an ultrasonic power of 50 kHz (Shoala 2020). To prepare the diluted solution, 3 or 1 mL of the prepared solutions was dissolved in one liter of distilled water. Characterization of nanomaterials using dynamic light scattering (DLS) The distribution and size of nano-glycyrrhizic acid ammonium salt nanoparticles (GAS-NPs), and salicylic acid nanoparticles (SA-NPs) were measured at room temperature using a dynamic light scattering method with the Zetasizer Nano ZS (Malvern Instruments, UK). Before measurement, 30 µl of nanoparticles were diluted with 3 ml of water at 25 °C. Particle size data was expressed as the Z-average of three different batches of nanoparticles.

For Transmission Electron Microscope investigation, a drop of the solution was applied to the carbon-coated copper grids (CCG) and allowed to evaporate at room temperature. Electron micrographs were taken at The Regional Center for Mycology and Biotechnology (RCMB) Al- Azhar University using a JEOL GEM-1010 transmission electron microscope operating at 80 kV [33].

The Fourier Transform Infrared Spectroscopy (FTIR) analysis of GAS-NPs and salicylic acid samples were conducted using a Thermo Scientific Nicolet iS50 FTIR spectrometer (Thermo Fisher Scientific, USA). The instrument was operated in attenuated total reflectance (ATR) mode with a diamond crystal plate. Spectra were collected in the mid-infrared region (4000–400 cm⁻¹) at a resolution of 4 cm⁻¹, with each spectrum being the average of 32 scans. Prior to sample analysis, a background spectrum was collected to eliminate atmospheric interference. The samples were placed directly on the ATR crystal and pressure was applied to ensure good contact.

X-ray Diffraction (XRD) patterns were obtained using a Bruker D8 Advance diffractometer (Bruker AXS, Germany) equipped with a Cu-Kα radiation source (λ = 1.5406 Å) operating at 40 kV and 40 mA. The samples were ground into fine powder and mounted on a silicon zero-background holder. Diffraction patterns were recorded in the 2θ range of 5–70° with a step size of 0.02° and a counting time of 1 s per step. The diffractometer was calibrated using a silicon standard before the measurements.

### Isolation and identification of the pathogen

#### Pathogen isolation

Tomato plants showing symptoms of Fusarium wilt were obtained. The infected root and stem portions (3–5 cm in length) were first surface-disinfected for two minutes with a 2% chlorine solution, following thorough washing with tap water. Before adding to PDA media (Potato, Dextrose, Agar) supplemented with 300 µg/ml streptomycin sulfate, the small pieces were carefully washed with sterile double-distilled water (ddH2O). They were then allowed to dry on filter paper under sterile conditions. The fungal cultures spent 7–10 days in an incubator maintained at 25 ± 2 °C [34].

#### DNA extraction and PCR assay

The genomic DNA of five isolates of *F. oxysporum* was extracted from single spore fungal cultures growing on a PDA medium using a DNeasy Plant Mini Kit from Qiagen-Hilden-Germany. To determine the isolate of *F. oxysporum*, PCR was performed with the specific primer sets uni-f and uni-r as described by [35]. The primers were synthesized as shown in Table S1. The uni primer set amplifies 670–672 bp fragments from all typical isolates of *F. oxysporum*. The PCR reaction mixture (25 µL) contained 18 µL of Master Mix (1.25 µL of 0.2 mM each dNTP mix, 5 µL Taq (10X), 2 µL (2.5 mM) MgCl_2_, 0.2 µL (1 U) Taq DNA polymerase, 9.55 µL distilled water), 1 µL (0.5 mM) of each primer (Uni, sp13, sp23, sprl, and ITS1-4), and 2 µL of genomic DNA. The PCR conditions for all primers were set at an initial denaturation temperature of 94 °C for 5 min, followed by 45 cycles of 94 °C for 1 min, annealing at 61 °C for 1 min and elongation at 72 °C for 2 min, with a final extension at 72 °C for 10 min [35]. All PCR reaction products were electrophoresed in a 1% agarose gel, stained with ethidium bromide, and visualized using UV light.

### Effect of different concentrations of GAS & SA nanoparticles on the growth of *Fusarium oxysporum in vitro*

A sterile disc containing a mycelial culture of the pathogen, aged for 7 days and measuring 4 mm in diameter, was placed at the center of 9-cm Petri dishes containing Potato Dextrose Agar (PDA) with a diameter of 9 cm. The impact of nanoparticles at four distinct concentrations (0.5, 1, 1.5, 3 ml/L) on the in vitro growth of *F. oxysporum* was assessed using a PDA medium. The control plates consisted solely of pathogen mycelial plugs. The plates were then incubated for 14 days at 25 ± 2 °C. Mycelial growth of the pathogen was measured (cm) on each plate, and the growth in the PDA medium supplemented with nanoparticles was compared with the growth of the pathogen in the control. Four replications of each treatment were tested, and mean values were calculated. The percentage reduction in colony diameter was calculated using the formula suggested by [36] as follows:$\:Reduction\%=\left[\frac{Gc-Gt}{Gc}\right]\times\:100$

Where: Gc = growth diameter in the control set. Gt = growth diameter in the treatment set.

### Effect of GAS-NPs & SA-NPs on the growth of selected beneficial bacterial strains in vitro

Four beneficial bacterial strains, previously isolated from potato soils and proven to have a positive impact, were selected: *Leclercia adecarboxylata* strain NM 114 (MT521702), *Pseudomonas putida* strain NM 115 (MT521730), *Enterobacter ludwigii* strain NM 2P2 (PQ349320), and *Bacillus marcorestinctum* strain NM31 (MT568523). *L. adecarboxylata* (Gram-negative) is known for growth promotion, phosphate and potassium solubilization, stress tolerance, and antimicrobial activities [37, 38]. *P. putida* (*fluorescent pseudomonads*) is recognized as a biocontrol agent for bioremediation and plant growth promotion [39]. *E. ludwigii*, a phosphate-solubilizing bacterium, enhances plant growth by solubilizing phosphorus and potassium, protecting crops, and improving soil health [40]. *Bacillus marcorestinctum*, a Gram-positive biocontrol agent, can quench quorum-sensing signals, reducing the severity of plant soft rot and potentially serving as a biological control agent for plant diseases [41].

The impact of GAS-NPs and SA-NPs on bacterial growth was investigated using the method published by Truong et al. [42], with adjustments. Four bacterial strains were grown on nutritional agar (NA) medium (Neogen NCM0110A and Agar Agar B&V) at 28 °C for 24 h. Suspensions of sterile phosphate buffer (PB 0.01) of each strain were made, and optical densities at 600 nm (OD600) were determined as follows: *B. marcorestinctum* (0.98), *E. ludwigii* (0.60), *L. adecarboxylata* (0.72), and *P. putida* (0.75). Forty-five nutrient broth tubes (10 ml each) were sterilized and divided into three treatment groups: one-third (15 tubes) were supplemented with SA-NPs to achieve a final concentration of 3 ml/L, another third with GAS-NP (3 ml/L), and the remaining third were left without any NPs. Bacterial suspensions (100 µL) were inoculated into three tubes representing each treatment group, with three tubes serving as negative controls (non-inoculated) for each group where the bacterial suspension was replaced by sterile phosphate buffer. Tubes were incubated at 28 °C for 24 h, and then optical densities at 600 nm (OD600) were determined for each tube, using negative controls (non-inoculated with bacteria) as blanks. Three blanks were considered to avoid nanoparticle interference: a blank without NPs, a blank supplemented with SA-NPs (3 ml/L), and a third supplemented with GAS-NPs (3 ml/L). Each inoculated treatment was compared with its corresponding blank.

### Effect of Nanoparticles on disease progress and growth parameters

#### Experimental design

Beliy Naliv-241 tomato seeds were surface-sterilized using a 0.5% sodium hypochlorite solution for 3 min, followed by rinsing with sterile distilled water. The seeds were then planted in seedling plug trays (3.4 × 3.4 × 5 cm), with 64 plugs per tray. These trays were maintained in greenhouse conditions at temperatures of 23 °C to 28 °C and relative humidity of 60–70%. After 21 days, when the tomato plants had developed three true leaves, the seedlings were transferred to 25-cm-diameter pots containing a standardized soil-sand mixture (80:20 ratio). As the design of the experiment (5 pots for each treatment) the tomato seedlings were treated with 50 ml/pot of concentration 1 or 3 ml/L drench of each nanoparticle separately or with 2.5 ml/L conventional fungicide (Kocide 2000) and planted in soil infested with FOL (isolate no. 2) at a concentration of 10^6^. Control plants received similar treatment with sterile distilled water and pathogen inoculation but without nanoparticles. Nanoparticles were applied before FOL inoculation (as a preventive measure) and seven days post-inoculation (as a curative measure), with appropriate control treatments. The experiment was repeated twice.

Following a period of 50 days, the extent of disease infestation was evaluated by determining the overall percentage of seedlings displaying symptoms associated with *Fusarium* wilt. These symptoms encompassed leaf yellowing and shedding, vascular discoloration, as well as alterations in plant height. Disease severity was calculated according to [34] Disease severity DS % = [Σ (n x i)]/ (N x I) x 100 Where: n = number of infected plants, i = the rate of infection, N = total number of plants, and I = the highest rate of infections.

### Microbial diversity among cultural microbial groupings

The impact of SA-NPs and GAS-NPs (1 ml/L and 3 ml/L, each) on tomato soil cultural bacteria and fungi was compared to the untreated control. Ten grams of soil following each treatment were suspended in 90 milliliters of sterile phosphate buffer (0.05 M), with three replications per treatment. The mixture was stirred for two hours at room temperature before being serially diluted. King’s medium B was used for fluorescent bacteria, along with other media for heterotrophic and copiotrophic bacteria [43]. Additionally, PDA was used to count total fungi. Incubation was made at 28 °C for 5–7 days.

#### Extracting total RNA and synthesizing cDNA

After 24 h, samples of young tomato leaves were collected from plants treated with GAS and SA nanoparticles at two different concentrations (1 ml/L and 3 ml/L), as well as control plants. The collected samples were crushed into free cells with liquid nitrogen. Using the Qiagen RNeasy^®^ Plant Mini kit (Cat. no. 51,304) and the manufacturer’s instructions, total RNA was extracted from tomato leaf samples. Following the manufacturer’s recommendations and using the gDNA Wipeout Buffer from the QuantiTect^®^ Reverse Transcription Kit, the gDNA contamination was eliminated from the isolated RNA. The isolated RNA was processed using the QuantiTect^®^ Reverse Transcription Kit (Qiagen, Cat. No. 205,311) to produce complementary DNA (cDNA). To prepare for future studies, the cDNA samples were kept at -20 °C.

#### Diferential expression analysis of the genes under investigation

Every cDNA sample underwent triplicate qRT-PCR as well as positive and negative cDNA template controls. For each qRT-PCR test, a final volume of 25 µl was obtained by adding 12.5 µl of SYBR Green PCR Master Mix (QuantiTect SYBR Green PCR Kit, Qiagen Cat. no. 204,143), 2.5 µl of cDNA, 0.3 µM of each forward and reverse primer given in Table 1, 1 µl of RNase inhibitor, and RNase-Free water to adjust the final volume. Following 10 min at 95 °C, 45 cycles of 95 °C for 20 s, followed by 60 °C for 20 s and 72 °C for 20 s, the reactions were then analyzed using an Agilent Technologies AriaMx Real-Time PCR System. Fluorescence was measured at the end of each cycle, and a 15-minute hold at 95 °C was made for the melting temperature analysis.

Fold change of gene expression ratios between treatment and control groups were calculated using the formula RQ = 2^−ΔΔC T^ [44], and all experimentally induced changes in the expression of the genes under study are expressed as n-fold variations from the respective controls. Suitable reference genes selection and validation to confirm accurate gene expression results is an essential step. We conduct initial validation before large-scale qPCR examination using two reference genes, *Actin* and Ribosomal protein L2 (*RPL2*) genes [45], data not discussed. *Actin* was the most stable gene, so we used it as an internal reference gene for qRT-PCR data normalization of the current study. The statistical analysis was carried out using Two-way ANOVA using SPSS, ver. 27 (IBM Corp. Released 2013). Data were treated as a complete randomization design according to [46]. Multiple comparisons were carried out applying Duncan test, the significance level was set at < 0.05.

**Table 1:** Gene names and sequences of quantitative real-time polymerase chain reaction (qRT-PCR) primers for gene expression profiling

Gene name	Primer Sequence (5’ ------ 3’)	Reference
*RAP-*F	AAAGAACCATCTGTGGCGTGTGAG	[26]
*RAP-*R	CGAATCTTGTAAGCGGCTTGGTCA	
*XET-2-*F	TGGAGGAGATTCTGCTGGTGTTGT	[28]
*XET-2-*R	TCTGTCTCCTTTGCCTCCTGTGAA	
*ACS-2-*F	TTCCATCACTGCAGCTTTGCTTCG	[28]
*ACS-2-*R	TTTGTTTGGGCCAGCTTCTCTCTC	
*PAL5-*F	GACAGCAGGAAGGAATCCAA	[30]
*PAL5-*R	CAACCAAATAGGGATTCGACA	
*LOXD-*F	TTGGCACCAAGTTCAGGCCC	[30]
*LOXD-*R	TGGACTTAAGCTAGTATTAG	
*PR1-*F	TGCCAAGACCGGTGGTAATTTC	[30]
*PR1-*R	TGCCCGCTAGCACATTGGT	
*Actin*-F (reference gene)	TTGCCGCATGCCATTCT	[28]
*Actin*-R (reference gene)	TCGGTGAGGATATTCATCAGGTT	
Ribosomal protein L2 *(RPL2)*-F (reference gene)	GTCATCCTTTCAGGTACAAGCA	[44]
Ribosomal protein L2 *(RPL2)*-R (reference gene)	CGTTACAAACAACAGCTCCTTC	

### Data analysis

All experiments were conducted in a completely randomized design. All parameters were tested for normality using the Shapiro-Wilk. The variables were generally normally distributed. Wilks’ Lambda Multivariate tests were used to evaluate the impact of treatments on different tested parameters. Post hoc, Bonferroni was used to compare the treatment groups to the control group (α = 0.05). The univariate analysis of variance was conducted to examine the effect of GAS-NPs & SA-NPs on the growth of the different bacterial strains *in vitro.* Dunnett’s t-test (two-sided) was conducted to compare different groups against the control group. All statistical analyses were performed using SPSS (Statistical Package for the Social Sciences, version 23).

## Results

### SA-NPs and GAS-NPs synthesis and characterization

**Fig. 1:**
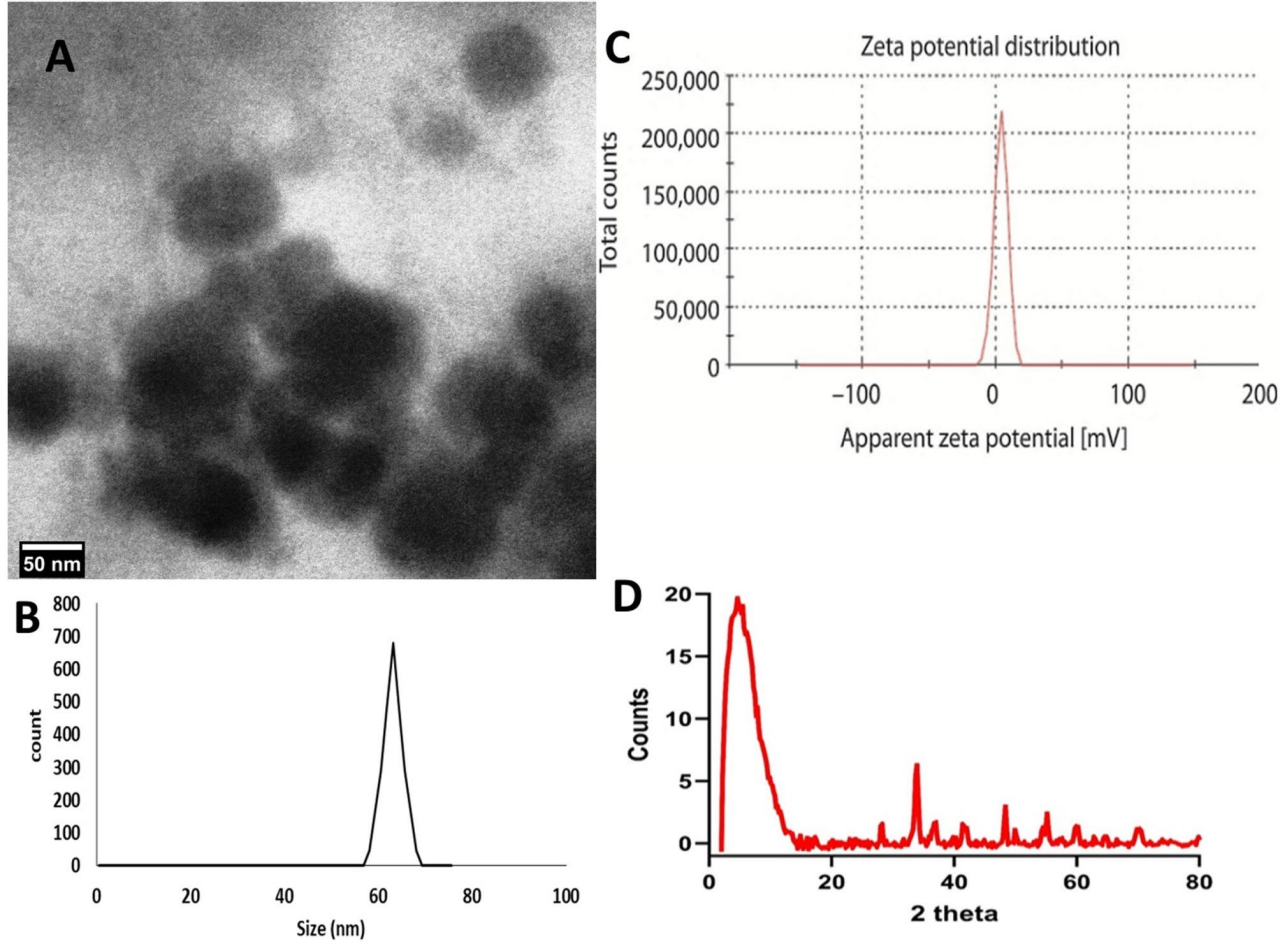
TEM **(A)**, Size Distribution **(B)**, Zeta Potential of glycyrrhizic acid ammonium salt nanoparticles **(C)**, and The X-ray diffraction (XRD) pattern of GAS-NPs sample **(D)**

**Fig. 2:**
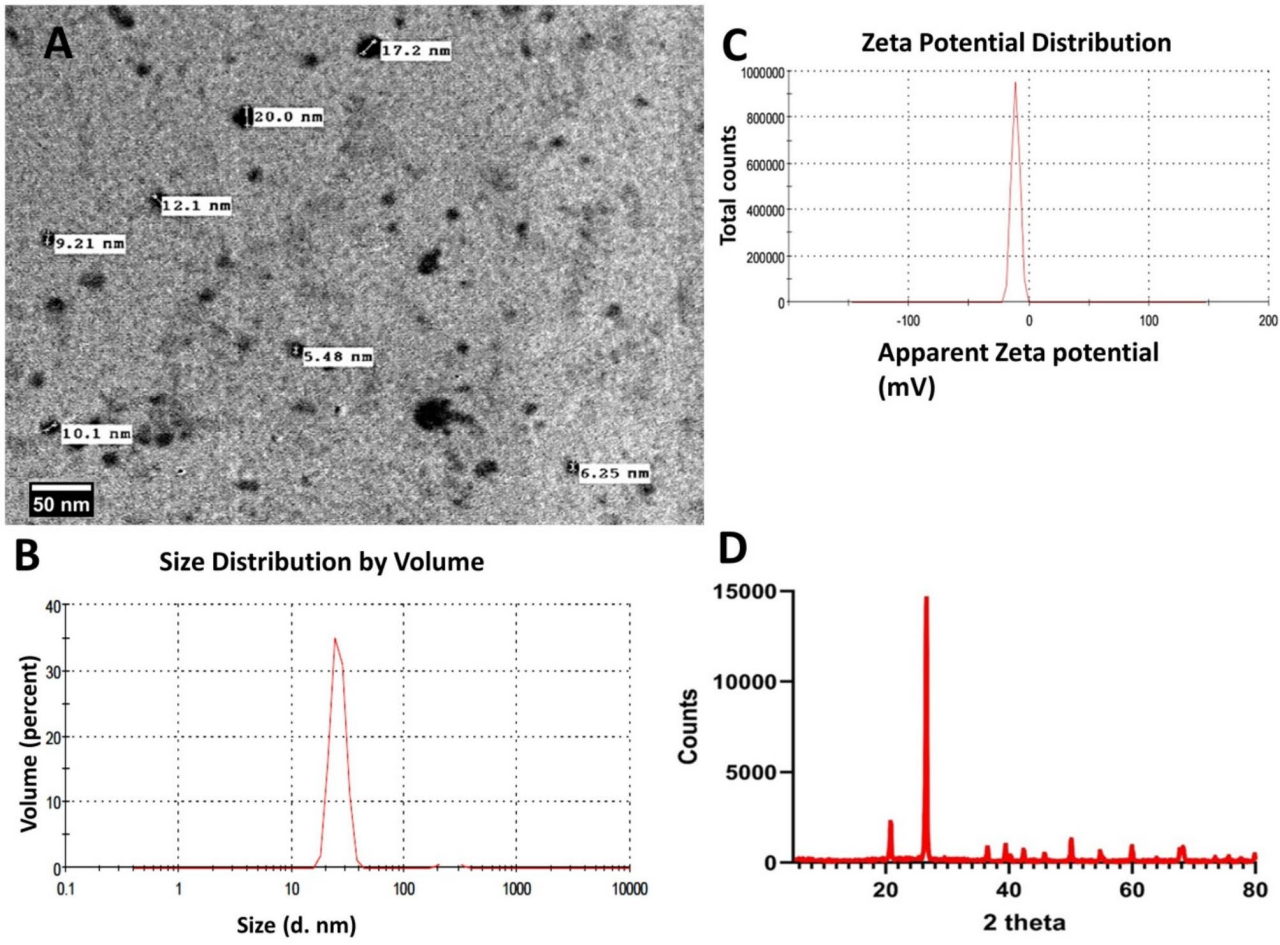
TEM **(A)**, Size Distribution **(B)**, and Zeta Potential of salicylic acid nanoparticles **(C)** and The X-ray diffraction (XRD) pattern of SA-NPs sample **(D)**

The Zetasizer Nano ZS (Malvern Instruments, UK) was utilized at ambient temperature to characterize salicylic acid and glycyrrhizic acid ammonium dispersion nanomaterials via dynamic light scattering (DLS). To measure, 30 µl of nanoparticles were mixed with 3 ml of water at 25 °C. Particle size was calculated using the Z-average of three distinct batches of nanoparticles. The mean size distribution was primarily 63 nm of glycyrrhizic acid ammonium salt dispersion nanomaterials (Fig. 1A-B) and 28 nm of salicylic acid nanoparticles, as shown in (Fig. 2A-B). The zeta potential is a useful indicator of colloidal dispersions’ stability. Colloids with a high zeta potential (positive) are electrically stable for glycyrrhizic acid ammonium nanoparticles (Fig. 1C). However, the zeta potential of salicylic acid colloids is negative (Fig. 2C). Transmission electron microscope (TEM) analysis showed that glycyrrhizic acid ammonium **(**Fig. 1A) and salicylic acid nanoparticles (Fig. 2A) were widely distributed and had an average size of < 100 nm. The TEM micrograph showed that glycyrrhizic acid ammonium nanoparticles had a circular morphology with an average size of 45–60 nm (Fig. 1A), whereas salicylic acid nanoparticles had a spherical shape with nano diameters ranging from 6 to 17 nm (Fig. 2A).

### XRD analysis of GAS-NPs sample

The X-ray diffraction pattern of the GAS-NPs sample (Fig. 1D) reveals an interesting crystallographic profile characterized by a combination of high-intensity peaks at low 2θ angles and several medium to low-intensity peaks distributed across the measured diffraction angle range (5–80° 2θ). The diffractogram is dominated by two prominent peaks at very low 2θ angles of 4.645° and 5.537°, corresponding to d-spacings of 19.009 Å and 15.948 Å, respectively. These peaks exhibit significantly higher intensities (1129 and 1056 counts) compared to other reflections in the pattern, suggesting the presence of a layered structure with large interplanar spacing.

The X-ray diffraction pattern of GAS-NPs sample showing its complex crystallographic profile. The diffractogram is characterized by distinctive low-angle features with a broad, intense peak centered around 5° 2θ (approximately 20 counts), indicating a layered structure with large interplanar spacing. This pattern is consistent with the presence of glycyrrhizin and other triterpene saponins that form lamellar arrangements. Secondary peaks of moderate intensity appear at approximately 33°, 48°, and 55° 2θ. The combination of crystalline peaks superimposed on a moderate background suggests a mixture of crystalline components within an amorphous matrix.

Beyond these dominant low-angle peaks, the pattern displays several medium-intensity peaks at 28.206° (d = 3.161 Å), 33.874° (d = 2.644 Å), 48.383° (d = 1.880 Å), and 55.083° (d = 1.666 Å). The remaining peaks at higher 2θ angles (59.995°, 64.648°, and 70.142°) show relatively lower intensities. Most peaks in the diffraction pattern appear sharp and well-defined, indicating good crystallinity of the constituent phases.

### XRD pattern description of Salicylic acid

The X-ray diffraction pattern of the salicylic acid sample (Fig. 2D) displays a highly crystalline profile with numerous sharp, well-defined peaks across the measured 2θ range (5–80°). Unlike the GAS-NPs sample, this pattern is characterized by the absence of dominant low-angle peaks and instead shows a distribution of high-intensity peaks in the mid-2θ range.

The X-ray diffraction pattern of salicylic acid showing characteristic crystalline features. The diffractogram displays exceptional crystallinity with numerous sharp, well-defined peaks across the 2θ range of 5–80°. The pattern is dominated by an extremely intense peak at approximately 25° 2θ (> 14,000 counts), with secondary prominent peaks at approximately 21° (2,000 counts), 39°, 46°, and 56°. The sharp, high-intensity peaks and excellent peak-to-background ratio indicate a highly ordered monoclinic crystal structure with minimal amorphous content, confirming the high purity of the pharmaceutical-grade salicylic acid sample.

The diffractogram is dominated by an exceptionally intense peak at approximately 28° 2θ, which shows the highest intensity (over 7000 counts) in the entire pattern. Other prominent peaks are observed at approximately 22° (intensity ~ 2000 counts), 40° (~ 800 counts), 43° (~ 500 counts), 50° (~ 1200 counts), and several peaks between 60–80° with moderate intensities (200–500 counts).

The peaks in the diffraction pattern are remarkably sharp and well-defined, indicating excellent crystallinity of the sample. The high peak-to-background ratio further suggests a highly ordered crystalline structure with minimal amorphous content.

### FTIR spectrum of glycyrrhizic acid ammonium salt nanoparticles

The FTIR spectrum of glycyrrhizic acid ammonium salt nanoparticles (Figure S2) reveals a complex molecular composition characteristic of plant-derived materials containing multiple bioactive compounds. The spectrum shows several distinctive absorption bands that can be attributed to various functional groups present in the phytochemical constituents of GAS-NPs, particularly glycyrrhizin, flavonoids, and other phenolic compounds.

The FTIR spectrum of GAS-NPs sample shows characteristic absorption bands. Key functional groups are identified by their corresponding wavenumbers (cm⁻¹): hydroxyl and N-H stretching region (3700–3000 cm⁻¹) with peaks at 3666.51, 3543.27, 3391.86, and 3302.67 cm⁻¹; C-H stretching at 2925.60 cm⁻¹; triple bond region (2250–2000 cm⁻¹) with multiple medium-intensity bands; aromatic C = C stretching at 1613.27 and 1530.47 cm⁻¹; C-H bending vibrations at 1441.66 and 1388.85 cm⁻¹; and the fingerprint region featuring C-O stretching at 1243.46 cm⁻¹, C-O-C stretching at 1147.16 cm⁻¹, and aromatic C-H out-of-plane bending at 763.99 cm⁻¹. The spectral pattern indicates the presence of glycyrrhizin, flavonoids, and various phenolic compounds characteristic of Glycyrrhiza species.

In the high-frequency region (3700–3000 cm⁻¹), multiple overlapping bands are observed. A sharp peak at 3666.51 cm⁻¹ corresponds to free hydroxyl (O-H) stretching vibrations, while broader bands at 3543.27 cm⁻¹ and 3302.67 cm⁻¹ are attributed to hydrogen-bonded O-H stretching of alcoholic and phenolic groups, which are abundant in glycyrrhizin and flavonoid compounds. The presence of a peak at 3391.86 cm⁻¹ suggests N-H stretching vibrations, possibly from amino acids or peptides present in the sample.

The C-H stretching region shows a prominent peak at 2925.60 cm⁻¹, characteristic of asymmetric stretching vibrations in methylene (-CH₂-) and methyl (-CH₃) groups, indicating the presence of aliphatic structures in the sample components.

Interestingly, the spectrum exhibits several medium-intensity bands in the 2250–2000 cm⁻¹ region (2216.09, 2200.04, 2163.32, 2135.92, 2094.63, and 2052.84 cm⁻¹), which may be attributed to various functional groups including C ≡ C stretching, C ≡ N stretching, and C = O stretching in anhydrides. These features suggest the presence of structurally diverse compounds in the GAS-NPs sample.

The carbonyl and aromatic region (1650–1400 cm⁻¹) displays strong absorption bands at 1613.27 and 1530.47 cm⁻¹, characteristic of C = C stretching vibrations in aromatic rings, which are prominent structural features in flavonoids and other phenolic compounds in GAS-NPs. The bands at 1441.66 and 1388.85 cm⁻¹ correspond to C-H bending vibrations in methyl and methylene groups, further confirming the presence of aliphatic moieties.

The fingerprint region (1300–500 cm⁻¹) contains several characteristic absorption bands that provide valuable structural information. The peak at 1243.46 cm⁻¹ is assigned to C-O stretching vibrations in phenolic compounds, while the band at 1147.16 cm⁻¹ indicates C-O-C stretching in ether linkages. A prominent peak at 1013.60 cm⁻¹ corresponds to C-O stretching vibrations in alcoholic groups. The aromatic C-H out-of-plane bending vibration at 763.99 cm⁻¹ further confirms the presence of aromatic structures in the sample.

### FTIR spectrum of Salicylic acid nanoparticles

The FTIR spectrum of salicylic acid (Figure S3) presents characteristic absorption bands that correspond to its known chemical structure (2-hydroxybenzoic acid). The spectrum shows distinct peaks that can be readily assigned to the key functional groups in salicylic acid: a carboxyl group (-COOH), a phenolic hydroxyl group (-OH), and an ortho-substituted benzene ring.

The FTIR spectrum of salicylic acid (2-hydroxybenzoic acid) with labeled absorption bands. Distinctive features include: free and hydrogen-bonded O-H stretching vibrations (3700–3000 cm⁻¹) with sharp peaks at 3622.93, 3399.08, 3353.18, and 3344.95 cm⁻¹; aromatic C-H stretching at 3081.57 and 3034.09 cm⁻¹; hydrogen-bonded carboxylic acid O-H stretching at 2728.68 and 2655.30 cm⁻¹; C = O stretching of carboxylic acid at 1626.43 cm⁻¹; aromatic C = C stretching at 1608.26 and 1512.99 cm⁻¹; C-O stretching of carboxylic acid and phenolic hydroxyl at 1299.42 and 1272.88 cm⁻¹, respectively; and the diagnostic C-H out-of-plane bending at 758.88 cm⁻¹ characteristic of ortho-substituted benzene rings. The spectrum confirms the chemical structure of salicylic acid with its carboxylic acid and phenolic hydroxyl functional groups.

The high-frequency region (3700–3000 cm⁻¹) exhibits multiple O-H stretching bands. A sharp peak at 3622.93 cm⁻¹ is attributed to free hydroxyl stretching, while broader bands at 3399.08, 3353.18, and 3344.95 cm⁻¹ correspond to hydrogen-bonded O-H stretching vibrations from both the carboxylic acid and phenolic hydroxyl groups. The position and intensity of these bands indicate significant hydrogen bonding, which is expected due to the intramolecular hydrogen bonding capability of salicylic acid.

The aromatic C-H stretching vibrations are observed at 3081.57 and 3034.09 cm⁻¹, while the aliphatic C-H stretching bands appear at 2955.75, 2921.74, 2870.44, and 2853.79 cm⁻¹. The characteristic O-H stretching bands of hydrogen-bonded carboxylic acid groups are seen at 2728.68 and 2655.30 cm⁻¹, which are typical for carboxylic acids and confirm the presence of the -COOH functionality. A strong absorption band at 1626.43 cm⁻¹ is assigned to C = O stretching vibrations of the carboxylic acid group, while bands at 1608.26 and 1512.99 cm⁻¹ correspond to C = C stretching vibrations in the aromatic ring. The C-H bending vibrations are observed at 1476.94, 1457.45, and 1376.06 cm⁻¹.

The C-O stretching region shows multiple bands, with peaks at 1299.42 cm⁻¹ (C-O stretching of carboxylic acid) and 1272.88 cm⁻¹ (C-O stretching of phenolic group) being particularly characteristic of salicylic acid. Additional C-O stretching vibrations are observed at 1241.49, 1211.13, and 1070.14 cm⁻¹.

The fingerprint region displays several distinctive bands, including C-H in-plane bending vibrations at 1169.22, 1144.39, and 1102.99 cm⁻¹, and C-H out-of-plane bending vibrations at 971.64, 842.50, 822.05, 803.22, 758.88, and 723.35 cm⁻¹. The band at 758.88 cm⁻¹ is particularly important as it is characteristic of 1,2-disubstituted benzene rings, confirming the ortho-substitution pattern in salicylic acid.

### Identification of *Fusarium oxysporum* using a polymerase chain reaction (PCR)-based technique:

The isolated fungus samples were PCR-amplified using uni-f and uni-r primers. This primer pair was used to directly amplify a 670–672 bp ribosomal (r) DNA fragment classified as belonging to *F. oxysporum*, extracted from mycelia. The effective amplification of the PCR analyses suggests that not all isolates were from the *F. oxysporum* species. The DNA fragment appeared to be identical in *F. oxysporum* isolates 2, 3, and 4, as shown in Figure S4A. The results show that only three of the five examined isolates (2, 3, and 4) are genetically similar to the *F. oxysporum* species. In contrast, no distinct *F. oxysporum* PCR amplicons were found in the remaining two isolates (Isolates 1 and 5). Among the three *F. oxysporum* isolates, the second isolate was identified as *F. oxysporum f. sp. lycopersici* race 1 using specific primers sp13, sp23, and sprl as shown in Table 2. The sequence was then uploaded to the gene bank under the accession number PQ578235 for Eukaryotic Nuclear rRNA/ITS/*Fusarium oxysporum* and validated by Uni-r (Fig. S4A and B).

**Table 2:** Three different *F. oxysporum* isolates amplified with specific primers to identify formae speciales and race-specific primers

	Uni	Sp13	Sp23	Sprl
2	+	+	-	-
3	+	-	-	+
4	+	-	-	+

### Effect of different concentrations of GAS & SA nanoparticles on the growth of *Fusarium oxysporum* (FOL) in vitro

The effects of SA-NPs and GAS-NPs on *F. oxysporum* (FOL), the causative agent of tomato wilt disease, are poorly understood. The impact of nanoparticles at four distinct concentrations (0.5, 1, 1.5, 3 ml/L) on the in vitro growth of FOL was assessed using a PDA medium containing the nanoparticles (Table 3). The growth of the pathogen in the control was compared with the growth in the nanoparticle-treated medium after 14 days. Univariate analysis of variance revealed a significant difference among different treatments (Type III sum of squares = 26.0, mean square = 3.2, F = 43.8, *P* < 0.001). The percentage reduction of the colony diameter for SA-NPs treatment at a 3 ml/L concentration was 37.8%. At the same time, it was 18.9% for GAS-NPs at the same concentration compared to the untreated control (*P* < 0.001). Also, SA-NPs treatment at 1.5 ml/L showed an 11% reduction, *P* = 0.01, according to the Bonferroni post-hoc test (Fig. S5). The data in Table 3 also indicated that all other concentrations were ineffective against FOL growth inhibition for both nanoparticles.

**Table 3:** The percentage reduction in the fungal colony diameter caused by SA and GA nanoparticles

NPs Concentrations	SA-NPs		GAS-NPs	
	Colony diameter cm (Mean ± SE)	Inhibition%	Colony diameter cm (Mean ± SE)	Inhibition%
0.5ml/L	8.7 ± 0.1	3.3	8.6 ± 0.15	4.4
1ml/L	8.6 ± 0.1	4.4	8.5 ± 0.12	5.6
1.5ml/L	8.0 ± 0.2*	11	8.4 ± 0.25	6.7
3ml/L	5.6 ± 0.2**	37.8	7.3 ± 0.15**	18.9
Control	9 ± 0.0	0	9 ± 0.00	0

*Significant difference as compared to the control, *P* = 0.01

** Significant difference as compared to the control, *P* < 0.001

Univariate analysis of variance revealed significant differences between different treatments (F = 43.8, *P* < 0.001)

### Effect of GAS-NPs & SA-NPs on the growth of selected beneficial bacterial strains in vitro

**Fig. 3:**
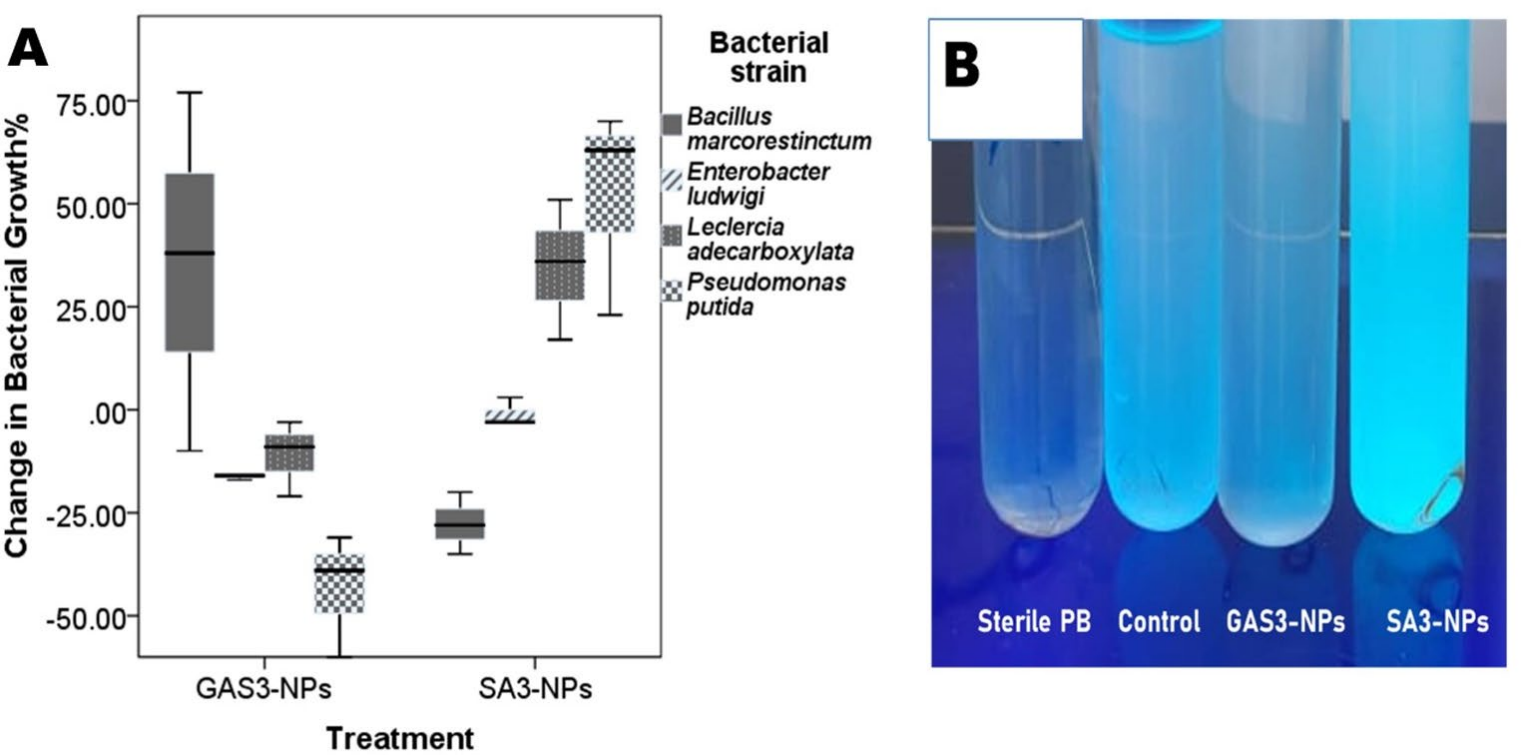
**A**) Change in bacterial growth for different strains after nanoparticle treatment compared to the untreated control. The optical density was determined after 24 h of inoculation and incubation in nutritional broth, while the control was left untreated with NPs. **B**) SA3-NPs caused a significant increase in *P. putida* growth, as shown under UV illumination, with a 51% increase determined at OD600. Three replicates were considered for each treatment. **Sterile PB: The nutrient broth (10 ml each) supplemented with 100 µL sterile PB Control: Inoculated with bacterial suspensions (100 µL) in sterile PB. **SA3-NPs: Supplemented with SA-NPs (3 ml/L) to achieve a final concentration of 3 ml/L and inoculated with bacterial suspensions (100 µL) in sterile PB. **GAS3-NPs: Supplemented with GAS-NPs (3 ml/L) to achieve a final concentration of 3 ml/L and inoculated with bacterial suspensions (100 µL) in sterile PB. **Three blanks were considered to avoid nanoparticle interference: a blank without NPs, a blank supplemented with SA3-NPs (3 ml/L), and a third supplemented with GAS3-NPs (3 ml/L). Each bacterial inoculated treatment was compared with its corresponding blank

Bacterial growth, as indicated by the difference in optical density (OD600), was as follows: Univariate analysis of variance revealed a significant difference in *E. ludwigii* growth among different treatments (Type III sum of squares = 0.006, mean square = 0.003, F = 5.2, *P* = 0.05). GAS-NPs caused a significant decrease only in *E. ludwigii* (17%, *P* = 0.05). Meanwhile, significant differences in the growth of *L. adecarboxylata* and *P. putida* among the treatments were observed (Type III sum of squares = 0.04, mean square = 0.02, F = 10.9, *P* = 0.01) and (Type III sum of squares = 0.21, mean square = 0.11, F = 17.0, *P* = 0.003), respectively. SA-NPs caused a significant increase in *L. adecarboxylata* (35%, *P* = 0.025) and *P. putida* (51%, *P* = 0.01), according to the Dunnett’s t (two-sided) post-hoc test (Fig. 3B). No other significant effects of the NPs were recorded (Fig. 3A).

### The effect of nanoparticles on disease progression and tomato growth metrics

#### Effect of the nanoparticles on tomato wilt severity

**Fig. 4:**
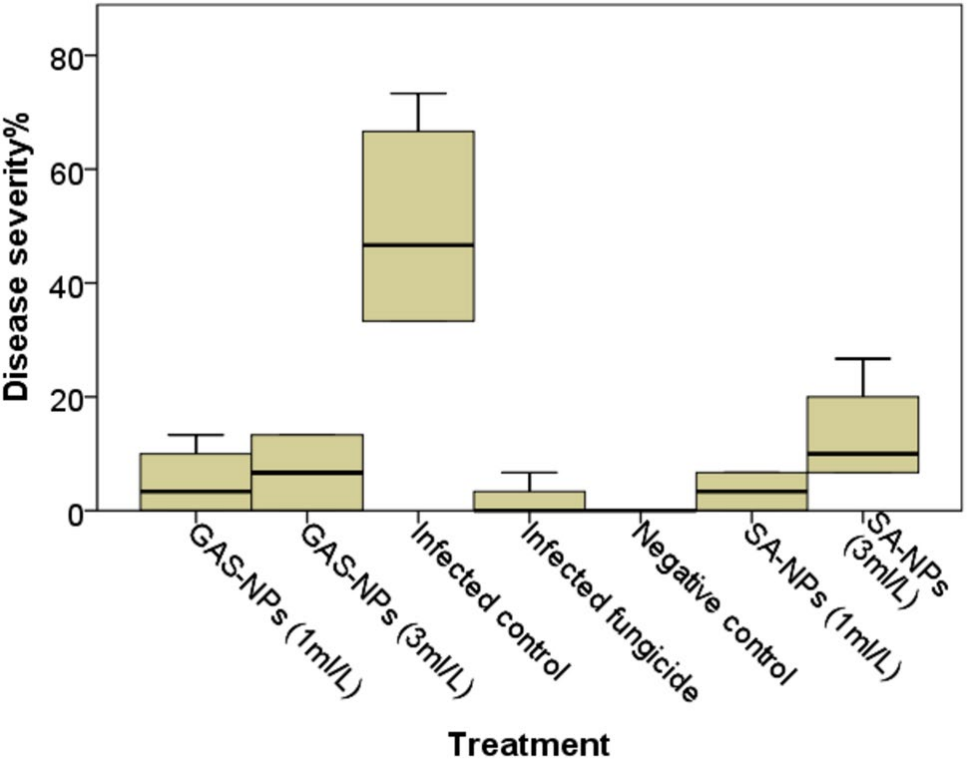
The effect of salicylic acid nanoparticles (SA-NPs) and glycyrrhizic acid nanoparticles (GAs-NP) at concentrations of 1 and 3 ml/L, compared to the standard fungicide Kocide 2000, on disease severity induced by *Fusarium oxysporum f.sp. lycopersici* (10^6^ conidia /g soil) after 80 days. Negative control: non-infested soil. All other treatments were used on plants grown in infected soil. *Univariate analysis of variance revealed a significant difference in disease severity among different treatments (Type III sum of squares = 7378.8, mean square = 1229.8, F = 14.0, *P* < 0.001)

Tomato-treated plants transplanted into infected soil exhibited severe symptoms as early as 12 days after infection, but plants treated with NPs or Kocide showed milder symptoms. Plants treated with 1 or 3 ml/L of NPs fared better than infected or Kocide-treated plants. Notably, plants treated with 1 ml/L of SA or GAS nanoparticles showed no significant difference from healthy plants (Fig. 4). Furthermore, the Wilks’ Lambda multivariate test demonstrated a substantial reduction in disease severity for the two nanoparticles (SA-NPs and GAS-NPs) at both doses (1 ml/L or 3 ml/L) as compared to the infected control (*P* < 0.001). Univariate analysis of variance revealed a significant difference in disease severity among different treatments (Type III sum of squares = 7378.8, mean square = 1229.8, F = 14.0, *P* < 0.001). The disease severity of the infected control group was 50%. SA-NPs reduced disease severity by 93% (*P* < 0.001) at the low dose (1 ml/L) and 73% at the high dose (3 ml/L). Similarly, GAS decreased 90% (*P* < 0.001) at the low dose and 87% (*P* < 0.001) at the high dose. The fungicide Kocide reduced disease severity by 97% (*P* < 0.001) according to the Bonferroni post-hoc test. There was no significant difference in disease severity between the two doses of each nanoparticle or the two nanoparticles themselves (Fig. 4). After 80 days, the amount of disease infestation was assessed by measuring the overall percentage of seedlings exhibiting Fusarium wilt symptoms. Symptoms included yellowing and dropping of leaves, vascular discoloration, and changes in plant height and other growth characteristics.

#### Effect of Nanoparticles on tomato growth parameters

**Fig. 5:**
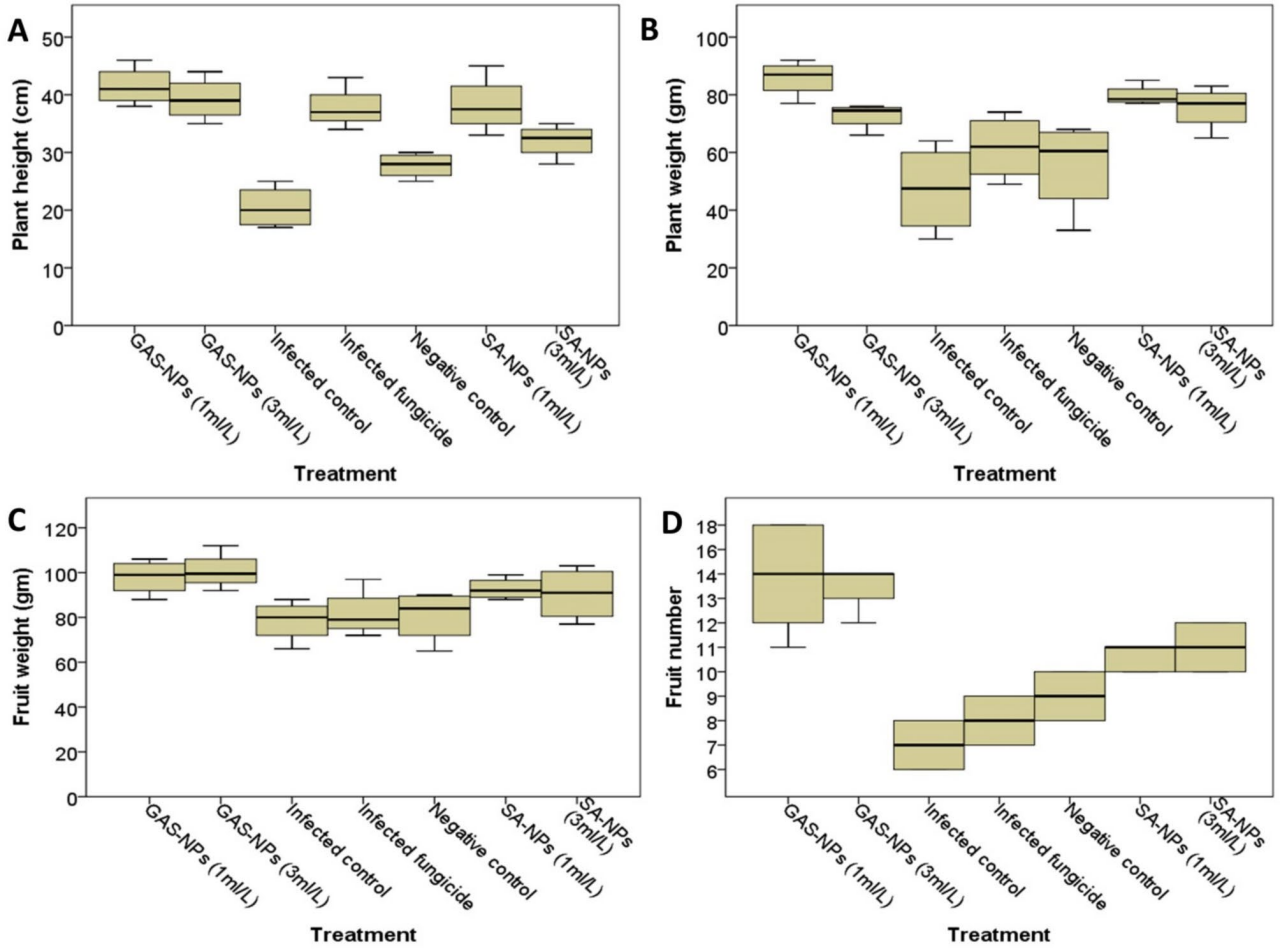
Vegetative growth parameters of tomato plants treated with salicylic acid and Glycyrrhizic acid ammonium salt nanoparticles at 1 and 3 ml/L, a standard fungicide (Kocide 2000) (infected), or untreated plants (negative) planted in infested soil

The effect of salicylic acid nanoparticles (SA-NPs) and glycyrrhizic acid nanoparticles (GA-NPs) on tomato plant growth metrics was studied in vivo. Tomato plants were treated with 1 or 3 ml/L of the nanoparticles or a conventional fungicide (Kocide 2000) and then planted in infested soil. Growth characteristics were evaluated after 80 days (Fig. 5). Plants treated with 1 or 3 ml/L nanoparticles had higher plant height, weight, fruit weight, and fruit number than those treated with Kocide or infected control (Fig. 5).

The study investigated the impact of various treatments on the vegetative parameters of tomato plants, including plant height (in centimeters), plant weight (in grams), fruit number, and total fruit weight (in grams). The data represent the mean of four dependent replicates, and the box plots were generated using IBM SPSS Statistics version 23. Multivariate tests (General Linear Model) revealed significant differences in plant height (Type III sum of squares = 1364.4, mean square = 227.4, F = 17.2, *P* < 0.001), plant weight (Type III sum of squares = 4628.4, mean square = 771.4, F = 7.1, *P* < 0.001), fruit weight (Type III sum of squares = 1865, mean square = 310.8, F = 3.4, *P* = 0.016), fruit number (Type III sum of squares = 185.4, mean square = 30.9, F = 13.3, *P* < 0.001).

The Bonferroni post-hoc analysis revealed that plants treated with 1 ml/L SA-NPs or GA-NPs differed significantly from healthy plants (Fig. S1). The research found no significant variations in vegetative parameters between infected and uninfected controls (Fig. 5). SA-NPs, at a low dose (1 ml/L), demonstrated an 87% increase (*P* < 0.001) in tomato plant height and a 69% increase (*P* = 0.004) in weight, with a trend of significant increase in fruit number (56%, *P* = 0.057) compared to the infected control. Meanwhile, at a high dose (3 ml/L), there was a 56% increase (*P* = 0.004) in tomato plant height, a 60% increase (*P* = 0.020) in weight, and a significant increase in fruit number (63%, *P* = 0.016). Both examined doses had no significant impact on fruit weight (Fig. 5).

GAS-NPs significantly increased tomato plant height (102%, *P* < 0.001) and weight (81%, *P* < 0.001), as well as fruit number (111%, *P* < 0.001) at a low dose (1 ml/L) compared to the infected control group. At a high dose (3 ml/L), tomato plant height increased by 91% (*P* < 0.001), weight increased by 54% (*P* = 0.037), and fruit number and weight increased significantly (96%, *P* < 0.001 and 28%, *P* = 0.05, respectively) (Fig. 5). There was no significant difference in the evaluated vegetative parameters between the two doses of each nanoparticle or the two nanoparticles themselves. Meanwhile, only GAS-NPs at high doses increased fruit weight significantly as compared to the infected control (Fig. 5).

Multivariate tests (General Linear Model) revealed significant differences in:

plant height (Type III sum of squares = 1364.4, mean square = 227.4, F = 17.2, *P* < 0.001),

plant weight (Type III sum of squares = 4628.4, mean square = 771.4, F = 7.1, *P* < 0.001),

fruit weight (Type III sum of squares = 1865, mean square = 310.8, F = 3.4, *P* = 0.016),

fruit number (Type III sum of squares = 185.4, mean square = 30.9, F = 13.3, *P* < 0.001).

**Fig. 6:**
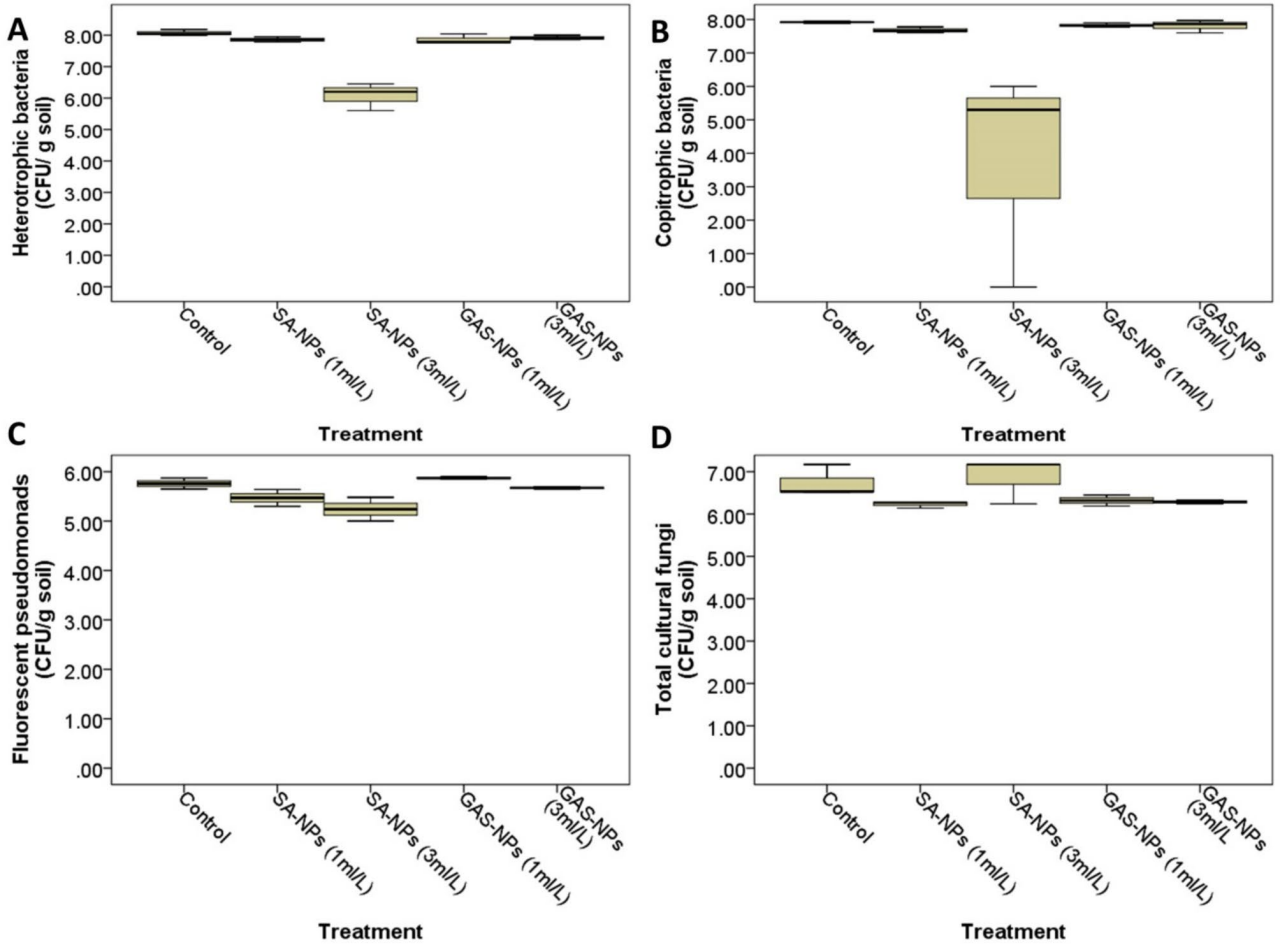
The Effect of Different Nanoparticles on Cultural Microbial Communities. This image depicts the effect on heterotrophic bacteria, copiotrophic bacteria, fluorescent pseudomonads (KB medium), and total fungi (PDA media). The nanoparticles studied were salicylic acid nanoparticles (SA-NPs) and glycyrrhizic acid nanoparticles (GAS-NPs) at two concentrations: 1 ml/L (SA1, GAS1) and 3 ml/L

Data in Fig. 6 summarizes the impact of different treatments on the cultural microbial community in tomato soil. There was a significant difference in the population of heterotrophic bacteria in tomato soil following different treatments (Type III sum of squares = 8.3, mean square = 2.1, F = 43.4, *P* < 0.001) (Fig. 6A). The Bonferroni post-hoc analysis reveals a significant decrease in heterotrophic bacteria with SA-NPs at high concentrations, from (8.07 ± 0.05) for the untreated control to (6.08 ± 0.25) (Log10 CFU ± SE) (*P* < 0.001). However, other treatments showed no significant differences compared to the control. There was also a significant difference in the population of copiotrophic bacteria in tomato soil following different treatments (Type III sum of squares = 11.8, mean square = 3.0, F = 82.1, *P* < 0.001) (Fig. 6B). A significant decrease in copiotrophic bacteria with SA-NPs at high concentration, from (7.96 ± 0.06) for the untreated control to (3.77 ± 1.89), (*P* < 0.001) was recorded. Additionally, Wilks’ Lambda multivariate test revealed a significant difference in the population of fluorescent pseudomonads in tomato soil following different treatments (Type III sum of squares = 0.76, mean square = 0.19, F = 9.5, *P* = 0.002). The Bonferroni post-hoc analysis revealed a significant decrease in the count of fluorescent pseudomonads (Fig. 6C), from (5.76 ± 0.06) for the untreated control to (5.24 ± 0.14) for SA-NPs at high concentration (*P* = 0.011). Meanwhile, no significant variation in total cultural fungi or Fusarium count was recorded (Fig. 6D).

#### Microbial biodiversity of the cultural microbial groups

Multivariate tests (General Linear Model) revealed significant differences in:

heterotrophic bacteria (Type III sum of squares = 8.3, mean square = 2.1, F = 43.4, *P* < 0.001).

copiotrophic bacteria (Type III sum of squares = 11.8, mean square = 3.0, F = 82.1, *P* < 0.001).

fluorescent pseudomonads (Type III sum of squares = 0.76, mean square = 0.19, F = 9.5, *P* = 0.002).

#### Gene expression profiling

Glycyrrhizic acid ammonium salt nanoparticles (GAS) and salicylic acid nanoparticles (SA) are systemic signal molecules that regulate the SAR of plants, according to quantitative examination of the mRNA levels of the defense-associated genes under study. *RAP*,* XET-2*,* ACS-2*,* PINII*,* PAL5*,* LOXD*,* and PR1* are tomato defense-associated genes that exhibited varying levels of expression when elicited by the two elicitors under study. These genes also displayed a 24-hour transcript accumulation.

**Fig. 7:**
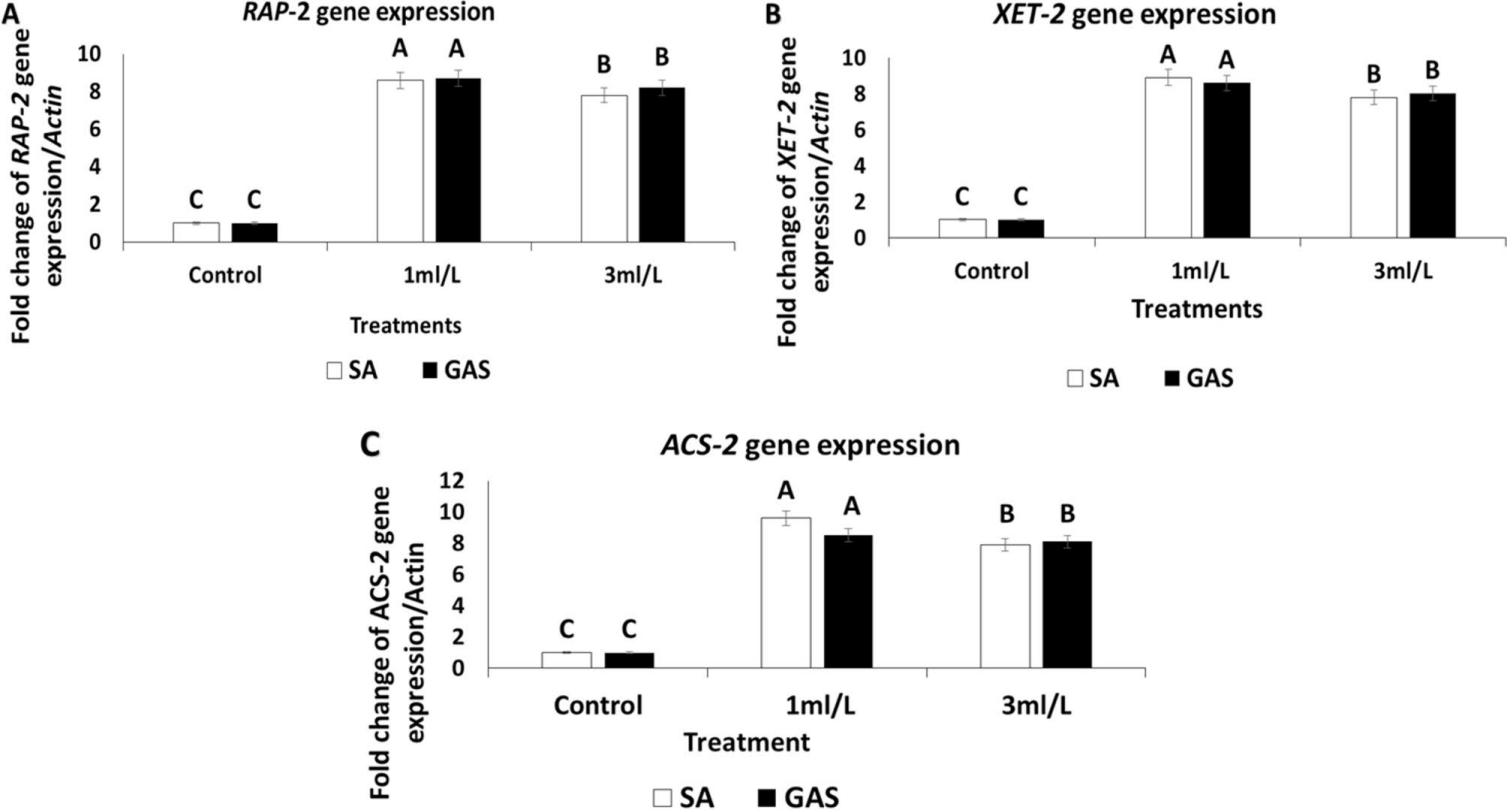
Differential expression analysis of genes linked to tomato defence mechanism. A: The *RAP-2*/*ACTIN* genes fold change, B: the *XET-2*/*ACTIN* genes fold change, and C: the *ACS-2*/*ACTIN* genes fold change after plant treatment with GAS-NPs and SA-NPs at two different concentrations (1 ml/L and 3 ml/L) as well as control, *Actin* gene, the most stable reference gene, was used as an internal reference gene

After being treated for 24 h with GAS-NPs and SA-NPs at two different concentrations (1 ml/L and 3 ml/L), tomato plants showed an increase in the expression of *RAP-2*,* XET-2*, and *ACS-2* genes. These targeted ethylene pathway genes (RAP-2, XET-2, and ACS-2) were upregulated in tomato plants sprayed with either GAS-NPs or SA-NPs in the corresponding amounts (Fig. 7A–C, **respectively**). For the *RAP-2*,* XET-2*, and *ACS-2* genes, treatment with 1 ml/L SA-NPs produced the largest amounts of mRNA transcripts, increasing 8.6-, 8.9-, and 9.6-fold, respectively.

**Fig. 8:**
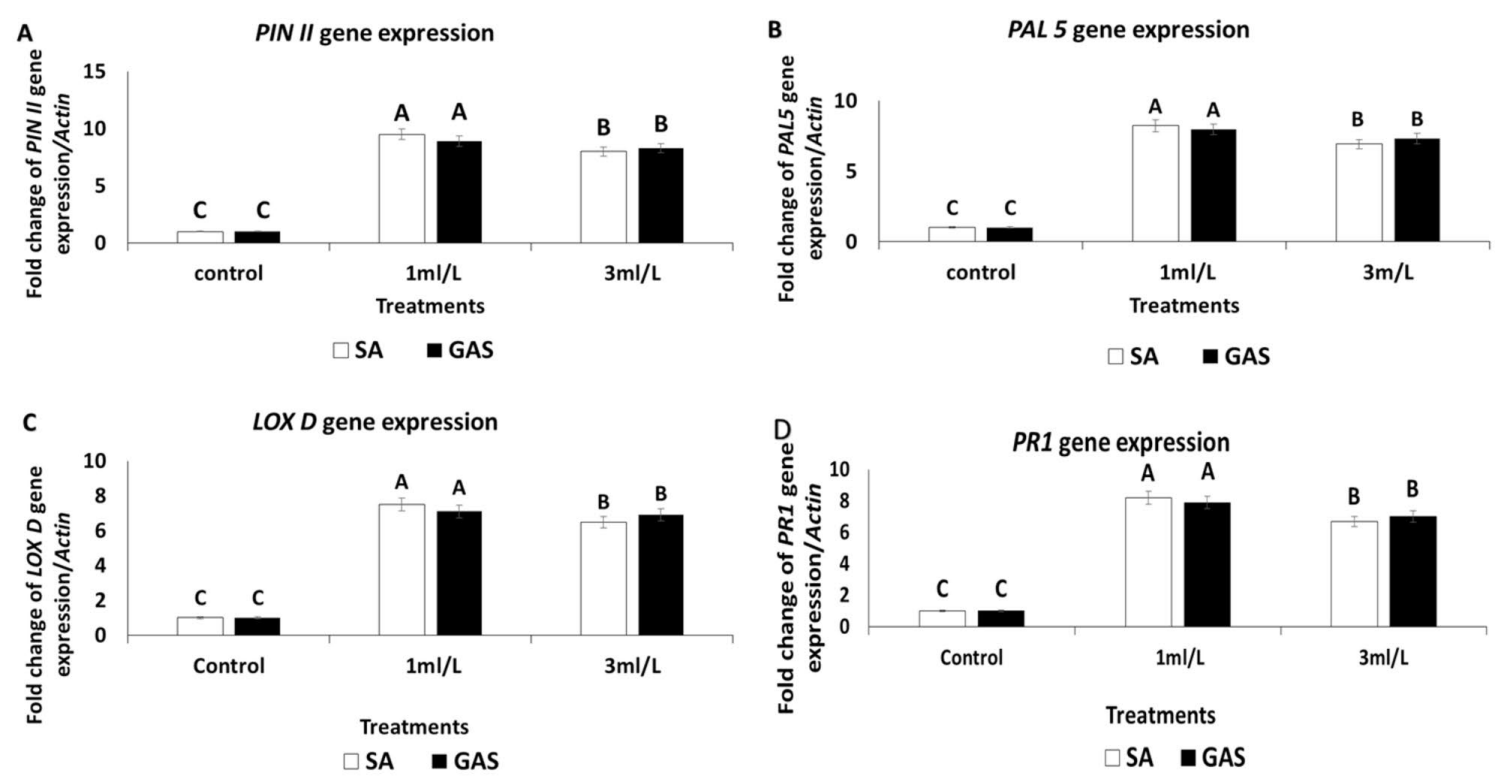
The impact of GAS-NPs and SA-NPs 1 ml/L and 3 ml/L as wll as control on gene expression of associated resistance genes using qRT-PCR analysis. **A**: *PINII*, **B**: *PAL5*, **C**: *LOX D*, and ** D**: *PR1* gene in treated tomato plant tissues. During data analysis, the *Actin* gene, the most stable reference gene, was used as an internal reference gene

The expression of *PINII*,* PAL5*,* LOXD*, and *PR1* genes involved in the jasmonate and salicylate pathways in tomato plants was also investigated in response to the two tested chemical inducers for SAR. According to the current study findings, the upregulation of defense-related genes could partially explain the improved management of tomato Fusarium wilt disease caused by *Fusarium oxysporum*, hence increasing productivity. All treatments used in this study resulted in increased mRNA levels of *PIN II*, *PAL 5*,* LOX D*, and *PR1* genes, as indicated in (Fig. 8A-D, respectively). As with the *PINII*,* PAL5*,* LOXD*,* and PR1* resistance genes, the highest levels of their expression were observed in tomato plant tissues treated with both GAS and SA-NPs at 1 ml/L. The highest mRNA transcript levels (9.5-, 8.2-, 7.5-, and 8.2-fold increase, respectively) for the same genes were observed after plant treatment with 1 ml/L SA-NPs. In general, tomato plants treated with all the chemical elicitors under investigation displayed greater expression signals of these genes in comparison to the control. Field treatment with 1 ml/L of both GAS and SA-NPs more strongly stimulated the expression of tomato resistance genes.

## Discussion

The FTIR spectra of salicylic acid nanoparticles (SA-NPs) and glycyrrhizic acid ammonium salt nanoparticles (GAS-NPs) reveal identical functional groups but different chemical compositions. GAS-NPs exhibit a more complex spectral pattern, with many overlapping bands and a wider range of absorption bands. The FTIR spectrum data are consistent with the expected phytochemical makeup of GAS-NPs. The prominent hydroxyl (O-H) stretching bands in the 3700–3300 cm⁻¹ region confirm the presence of multiple hydroxyl-containing compounds, which is consistent with the polyphenolic nature of major bioactive components in GAS-NPs, particularly glycyrrhizin (a triterpene glycoside) and various flavonoids. The strong absorption bands in the aromatic region (1613.27 and 1530.47 cm⁻¹) provide evidence for the flavonoid content of the GAS-NPs, as these compounds possess characteristic aromatic ring structures. The presence of C-O stretching vibrations at 1243.46 cm⁻¹, typical of phenolic compounds, further supports this interpretation. The C-O-C stretching vibration at 1147.16 cm⁻¹ is particularly significant as it indicates glycosidic linkages, which are present in glycyrrhizin—the principal active compound in GAS-NPs that consists of a triterpene aglycone (glycyrrhetinic acid) linked to a disaccharide unit. The C-H stretching and bending vibrations (2925.60, 1441.66, and 1388.85 cm⁻¹) suggest the presence of the triterpene backbone, which contains multiple methyl and methylene groups. Salicylic acid, on the other hand, presents a more defined spectrum with sharper peaks, characteristic of a pure compound with a specific chemical structure. The presence of C-O-C stretching vibrations in GAS-NPs suggests glycosidic linkages, which are absent in salicylic acid. The carbonyl absorption in salicylic acid is more distinct compared to GAS-NPs, where carbonyl absorptions are either weaker or overlap with other bands. The fingerprint region of salicylic acid shows a characteristic pattern of C-H out-of-plane bending vibrations, particularly the band at 758.88 cm⁻¹, which is diagnostic of its ortho-substituted benzene ring structure.

The XRD patterns of GAS-NPs and SA-NPs samples reveal significant differences in their nature and composition. The salicylic acid sample has higher crystallinity, with sharper peaks and a better peak-to-background ratio, indicating its pure nature. The GAS-NPs sample contains a complex mixture of compounds with varying degrees of crystallinity. The salicylic acid pattern has a more uniform distribution of peaks across the 2θ range, with the most intense peaks in the mid-angle region. The salicylic acid pattern corresponds to a single crystalline phase, while the Glycyrrhiza pattern suggests multiple crystalline phases. The salicylic acid sample has a larger average crystallite size (~ 85–95 nm) compared to the Glycyrrhiza sample (~ 45–50 nm), indicating better-developed crystalline domains in the pharmaceutical compound.

*Fusarium oxysporum f*.sp. *lycopersici* (FOL) wilt is one of the most severe diseases affecting tomato productivity in Egypt, particularly FOL race 1 and FORL. Recent advancements in nanotechnology have demonstrated significant potential in managing plant diseases. The present study was conducted to evaluate the efficacy of salicylic acid nanoparticles (SA-NPs) and glycyrrhizic acid ammonium salt nanoparticles (GAS-NPs) against *Fusarium oxysporum* (FOL race 1) in both in vitro and in *vivo* conditions. Additionally, the study assessed the impact of GAS-NP & SA-NP on the growth of beneficial bacterial strains *in vitro.* In this research, SA-NPs and GAS-NPs have been proven to improve plant growth characteristics considerably. While the NPs had no direct effect on certain beneficial bacteria (such as PGPR and biocontrol agents) in vitro. SA-NPs were shown to stimulate the growth of some PGPR bacterial strains. These findings emphasize the need for further research into the combined impact of particular nanoparticles and selected beneficial bacteria. Notably, combining nanoparticles and PGPR was proven to be considerably more effective in boosting plant development than their solo applications [47]. Advanced nanotechnology attempts to develop novel nanomaterials for specific stresses. That is how agronomy, microbiology, and nanotechnology may work together to significantly improve agricultural stress management for more robust, efficient, and sustainable farming [48].

When tested in vitro, both SA-NPs and GAS-NPs nanoparticles revealed the ability to inhibit *F. oxysporum* growth at increased levels (3 ml/L), with SA-NPs outperforming GAS-NPs. In this study, increasing the concentration of the nanoparticles used improved their inhibitory activity against the pathogen in vitro. This is consistent with previous findings by [49], who reported that increasing the concentration of both chitosan and chitosan nanoparticles reduced radial development, spore production, and sclerotia germination in all tested fungi. Nanomaterials are efficient antimicrobial agents due to their large surface area-to-volume ratio and distinct chemical and physical properties, which improve their interaction with pathogens and capacity to infiltrate cells.

However, in greenhouse conditions, high doses of SA-NPs were found to be less efficient in disease suppression when compared to all other treatments.

These findings were associated with a substantial decline in the overall bacterial population, as seen by significant declines in heterotrophic bacteria, copiotrophic bacteria, and fluorescent pseudomonads following the administration of a high dose of SA-NP. Copiotrophic and heterotrophic bacteria, as well as fluorescent pseudomonads, typically contribute to the control of soil-borne diseases through processes such as nutrient competition, antimicrobial chemical production, and plant defense induction. A decline in these beneficial bacteria can weaken their antagonistic effects, making the soil more vulnerable to Fusarium infections [50]. Fluorescent pseudomonads are recognized for producing antibiotics and siderophores that prevent diseases from growing. A reduction in their population can limit the soil’s natural disease suppression capacity, rendering plants more vulnerable to Fusarium wilt and other diseases [51]. A healthy soil microbial community is diverse and balanced, which improves soil health and plant growth. When beneficial bacteria are reduced, the equilibrium is disturbed, allowing diseases like Fusarium to spread [52]. Meanwhile, while the high dose of SA-NPs significantly reduced culturable bacteria in the tomato rhizosphere, particularly fluorescent pseudomonads, it enhanced bacterial growth of *L. adecarboxylata* and *P. putida in vitro*, which suggests no direct influence on bacterial growth. Soil is a complex and dynamic habitat, whereas in vitro conditions are highly controlled and specific. The results may represent the interaction between SA-NPs and the tomato rhizosphere, potentially impacting bacterial colonization indirectly. Nanoparticles may affect soil pH and nutrient availability, interrupting the microbial community [53, 54]. SA-NPs, for example, may modify soil pH because salicylic acid’s acidic effect favors certain strains over others and influences organic matter mineralization. It has been demonstrated that salicylic acid-loaded selenium nanoparticles suppress bacterial biofilm formation by *Escherichia coli*, *Pseudomonas aeruginosa*,* Bacillus subtilis*, and *Staphylococcus aureus* [55]. Furthermore, even low dosages of some nanoparticles, such as AgNPs, applied over time caused substantial decreases in soil microbiota, nitrogen-fixing bacteria, and microbial activity. Some strains’ survival may promoted in the presence of SA-NPs due to enhanced nutrient availability, surface interactions, or other conditions in a more dynamic soil environment. As a result, more research is needed addressing the potential long-term gradual release of nanoparticles, which might impact soil health [56]. Lebeis et al. [57] studied how SA alters the colonized bacterial population by influencing root colonization by certain bacterial families. Seitz et al. [58] also investigated the effects of root exudate composition on the soil microbiota.

Profiling of the studied tomato resistance genes expression using qRT-PCR revealed that the studied gene expression was more strongly induced by field treatment with 1 ml/L of both GAS-NPs and SA-NPs. Tomato plants sprayed with either GAS-NPs or SA-NPs at their respective amounts showed upregulation of the targeted ethylene pathway genes (*RAP*,* XET-2*,* and ACS-2*) (Fig. 7A–C, respectively). Under field circumstances, plant treatment with 1 ml/L SA-NPs resulted in the highest quantities of mRNA transcripts for *RAP*, *XET-2*, and *ACS-2* genes, increasing 8.6-, 8.9-, and 9.6-fold, respectively. When compared to the control, tomato plants treated with all the investigated chemical elicitors generally showed higher expression signals of these genes. The results of Herman et al. [59] and El-Garhy et al. [30], who found that SA enhanced SAR and promoted the synthesis of secondary metabolites associated with plant defence, were consistent with our findings of the differential expression of the targeted defence genes. They reported that SAR was distinct from other plant defense responses by the indigenous and systemic stimulation of specific pathogenesis-related genes (PR genes).

Additionally, the RAP gene’s stimulation in this study was consistent with the findings of Phukan et al. [60] and El-Garhy et al. [30], who showed that the ethylene response factor gene (RAP) was a crucial component of these signalling cascades because it controlled the expression of numerous genes linked to development and stress response via various mechanisms. Furthermore, this study’s *XET-2* gene activation was consistent with Catala et al. [61]’s findings, which proposed that the XET-2 gene encoded xyloglucan endotransglycosylase.

The xyloglucan endo-cleavage polymers and the subsequent transfers of the newly formed reducing ends to other polymeric or oligomeric xyloglucan molecules are also stimulated by this gene.

Therefore, by rearranging the load-bearing xyloglucan cross-links between cellulose microfibrils, XET protein action provides a possible tool to accomplish regulated wall relaxation during turgor-driven expansion. However, the ACS–2 gene’s regulation in this study was consistent with Yang et al.‘s [62] findings, which stated that ACS–2 was a gene involved in ethylene production. Furthermore, the expression of genes linked to pathogenesis that are part of the tomato plant’s defence mechanism has greatly increased its resistance to a variety of diseases, according to Alexandersson et al. [63].

The *PINII* gene’s differential expression in this study was consistent with the findings of Turra and Lorito [64] and El-Garhy et al. [30], who looked into how the PINII gene was expressed in response to diverse environmental conditions, injuries, and field applications.

Bacterial and viral infections, insect and nematode attacks, and the use of fungal resistance elicitors can potentially activate the *PINII* gene. However, aspirin and SA can suppress *PINII* gene activation caused by damage, jasmonic acid, or systemin. Furthermore, the study’s observation of *PAL5* gene stimulation was consistent with Chandrasekaran and Chun’s [65] findings that phenyl ammonia lyase (PAL) is a crucial enzyme in the metabolism of phenylpropanoid, which results in the synthesis of defensive compounds (lignins, coumarins, flavonoids, and phytoalexins). PAL is an essential protein for both plant growth and pathogen defence. Pathogenesis-related proteins (PR), including PR-1 protein, are elevated in the generated systemic resistance [30]. The results of Safaie-Farahani and Taghavi [66], who suggested that lipoxygenases (LOXs) may function as signaling molecules involved in structural and metabolic changes in plants, leading to resistance against the pathogen, were also supported by the upregulation of the LOXD gene seen in this study. The application potential of the Tom LoxD gene for crop protection against insects and pathogens was emphasized by **Yan et al.** [67],.

The application of these natural materials should be undertaken with caution, and additional research is imperative to enhance their effectiveness while minimizing any adverse impact on the indigenous microbial community.

## Conclusion and future perspectives

Natural nanomaterials can efficiently manage phytopathogens while remaining environmentally friendly. However, more research is needed to improve effective nanoparticle dosages, taking into account the balance between successful disease control and long-term preservation of the beneficial complexity of soil microbial biodiversity using NGS analysis.

## Electronic supplementary material

Below is the link to the electronic supplementary material.

Supplementary Material 1

## Data Availability

The datasets generated and/or analyzed during the current study are available in ACCESSION NUMBER TO DATASETS”.The sequence was then uploaded to the gene bank under the accession number PQ578235 for Eukaryotic Nuclear rRNA/ITS/Fusarium oxysporum and validated by Uni-r in current manuscript Fig S4https://www.ncbi.nlm.nih.gov/nuccore/PQ578235.1/.

## Abbreviations

ROS: Reactive Oxygen Species
SA-NPs: Salicylic Acid Nanoparticles
GAS-NPs: Glycyrrhizic Acid Ammonium Salt Nanoparticles
DLS: dynamic light scattering
FOL: *Fusarium oxysporum f*.sp. Lycopersici
PINII: Genes Proteinase Inhibitor II
PAL5: Phenylalanine Ammonia-Lyase 5
LOXD: Lipoxygenase D
XET-2: Xyloglucan Endotransglucosylase 2
ACS-2: Catalytic Hydrolase-2
RAP: ethylene-responsive transcription factor 3
PR1: pathogenesis-related protein 1
PR: Pathogenesis-related proteins
