# Supplementary material for: The efficacy of nanoparticles on soil microbial biodiversity and the prevention of Fusarium wilt disease (Fusarium oxysporum f.sp. lycopersici)
Source: BMC Microbiol. 2025 May 19;25:305. doi: 10.1186/s12866-025-04022-3 (PMC12087229; doi:10.1186/s12866-025-04022-3)
Supplement: Supplementary file 1 — Supplementary Material 1 [file 12866_2025_4022_MOESM1_ESM.docx]

**Supplementary material**

**Supplementary materials**

**Table S1: Specific primer sets of PCR analyses:**

| Primer sets | Primer Sequence (5’ ------ 3’) Fragment (bp) | Position |
| --- | --- | --- |
|  |  | 672-bp, ff. sp. *Lycopersici* and *radicis-lycopersici* |
| uni-f | ATCATCTTGTGCCAACTTCAG | nt 170–190 of Saitama ly1 pg1 |
| uni-r | GTTTGTGATCTTTGAGTTGCCA | nt 841–820 of Saitama ly1 pg1 |
|  |  | 445-bp, f. sp. *Lycopersici* race 1 and 3 |
| Sp13-f | GTCAGTCCATTGGCTCTCTC | nt 896–915 of Saitama rly *pgx4* |
| Sp13-r | TCCTTGACACCATCACAGAG | nt 1340–1321 of Saitama rly pgx4 |
|  |  | 518-bp, f. sp. *Lycopersici* race 2 and 3 |
| Sp23-f | CCTCTTGTCTTTGTCTCACGA | nt 240–260 of Saitama ly1 *pg1* |
| Sp23-r | GCAACAGGTCGTGGGGAAAA | nt 757–738 of Saitama ly1 pg1 |
|  |  | 947-bp, f. Sp. *Radicis-lycopersici* |
| sprl-f | GATGGTGGAACGGTATGACC | nt 385–404 of Saitama rly *pgx4* |
| sprl-r | CCATCACACAAGAACACAGGA | nt 1330–1310 of Saitama rly pgx4 |
| ITS1-4 | 5′-TCCGTAGGTGAACCTGCGG-3'  5′-TCCTCCGCTTATTGATAGATAC-3' | 500-650 bp |


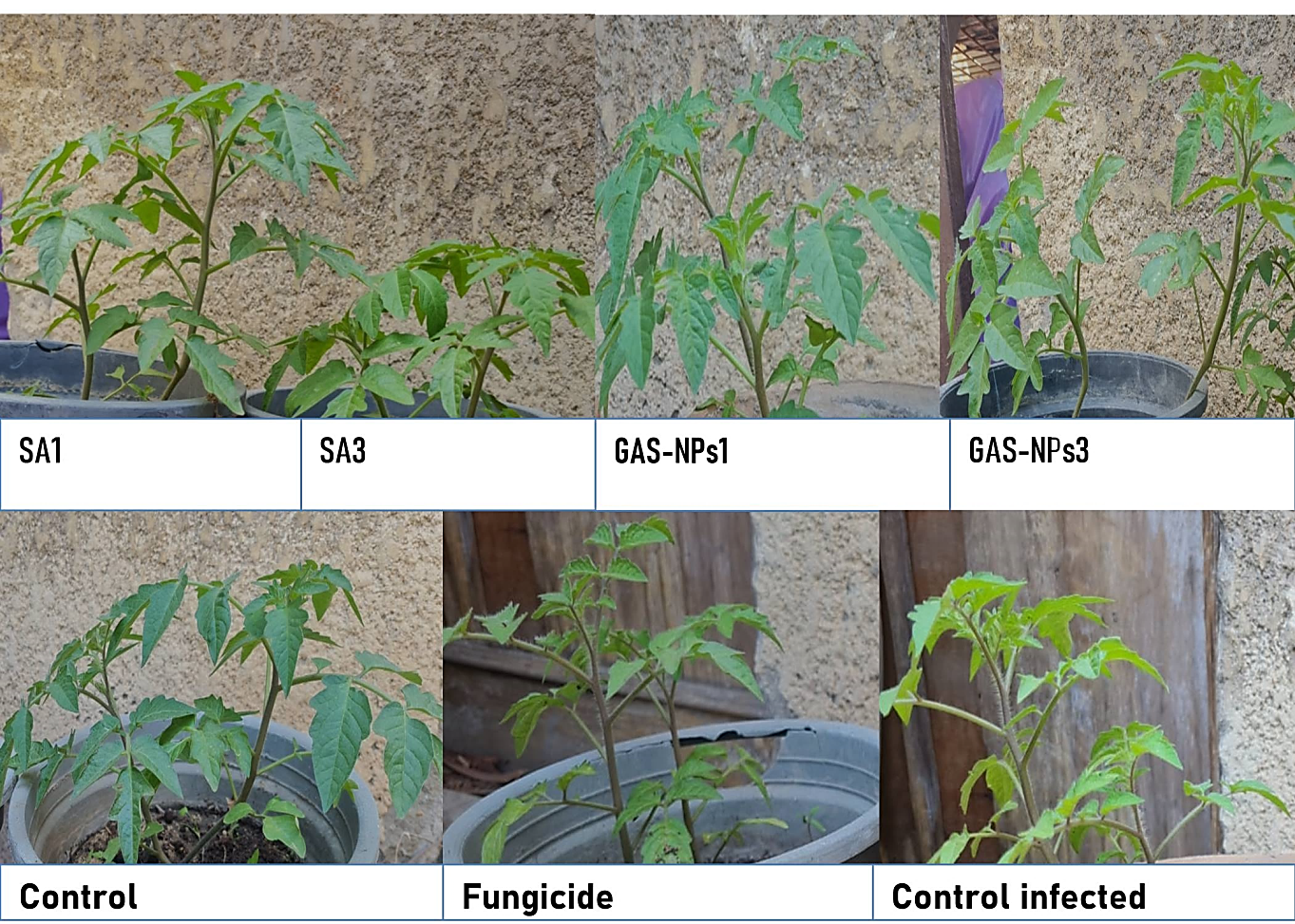


**Fig. S1** Effect of SANPs and GASNPs at concentration 1and 3ml/L on tomato plants were grown in infested soil with Fusarium oxysporum race 2 in compared with positive control, negative control and Fungicide.


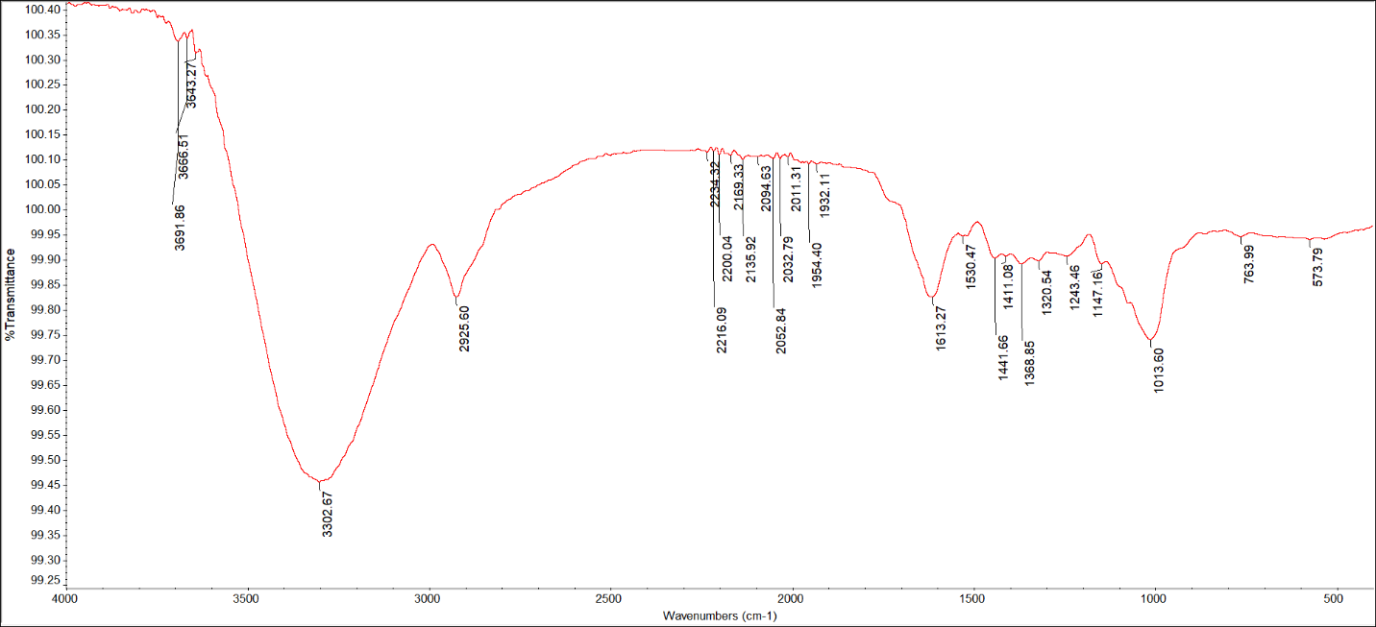


**Fig. S2. FTIR Spectrum of** GAS-NPs **(4000-400 cm⁻¹)**


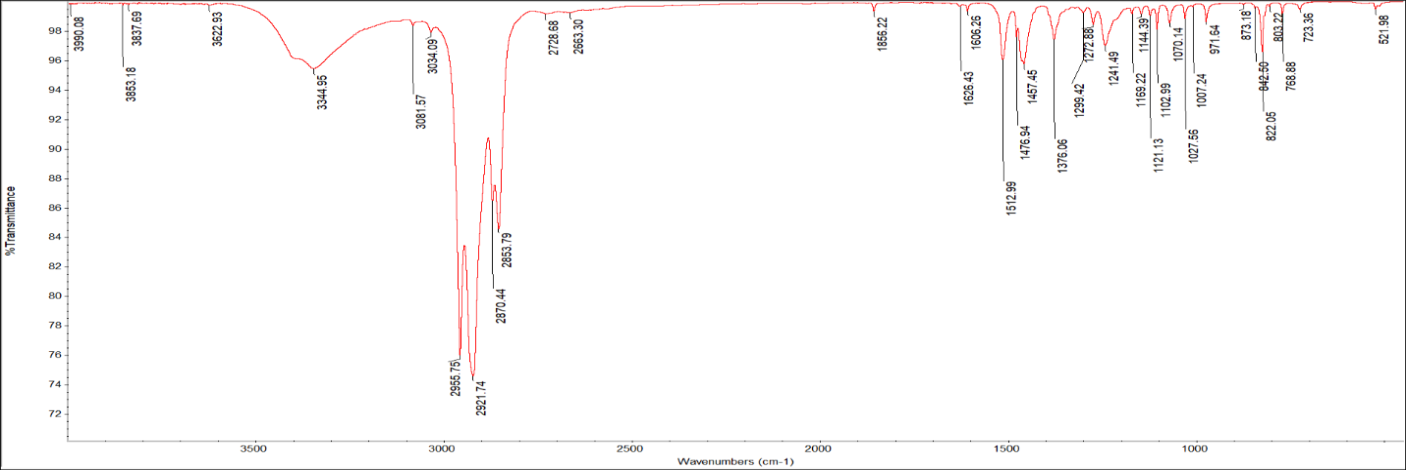


**Fig. S3. FTIR Spectrum of Salicylic Acid (4000-400 cm⁻¹)**


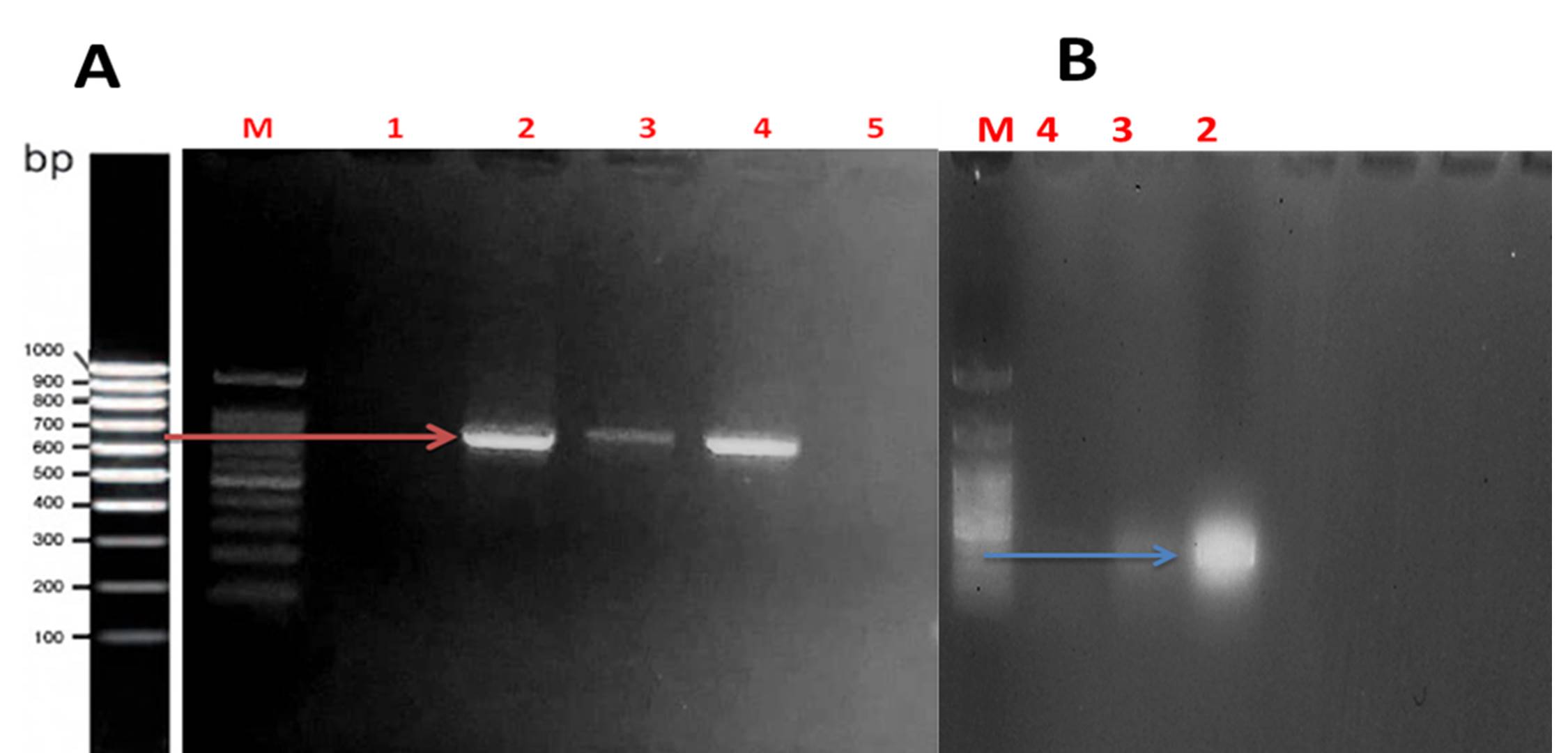


**Fig. (S4): PCR gel electrophoresis of five distinct fungal isolates (1, 2, 3, 4, 5) amplified with uni-f and uni-r unique primers. A) The uni primer set amplifies 670-700 bp fragments for typical *F. oxysporum* isolates, with a ladder of 100 bp (M). B) The sp13 primer set amplifies 400-500 bp fragments for typical** *F. oxysporum f. sp. lycopersici* race 1**, with a ladder of 100 bp.**


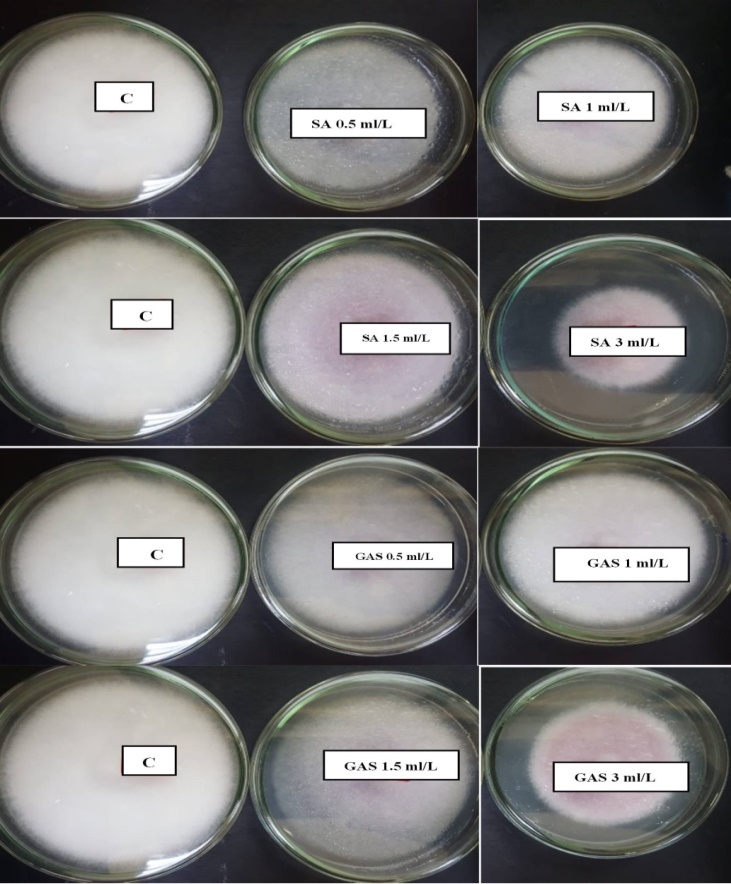


**Fig. (S5)** Effect of Four different dosages (0.5 ml/L, 1 ml/L, 1.5 ml/L, and 3 ml/L) of (SA-NPs): Salicylic acid nanoparticles and (GAS-NPs): Glycyrrhizic acid ammonium salt nanoparticles on mycelia growth of *F. oxysporium* on PDA medium.
